# Coronavirus replication–transcription complex: Vital and selective NMPylation of a conserved site in nsp9 by the NiRAN-RdRp subunit

**DOI:** 10.1073/pnas.2022310118

**Published:** 2021-01-20

**Authors:** Heiko Slanina, Ramakanth Madhugiri, Ganesh Bylapudi, Karin Schultheiß, Nadja Karl, Anastasia Gulyaeva, Alexander E. Gorbalenya, Uwe Linne, John Ziebuhr

**Affiliations:** ^a^Institute of Medical Virology, Justus Liebig University Giessen, 35392 Giessen, Germany;; ^b^Department of Medical Microbiology, Leiden University Medical Center, Leiden 2300 RC, The Netherlands;; ^c^Department of Biomedical Data Sciences, Leiden University Medical Center, Leiden 2300 RC, The Netherlands;; ^d^Faculty of Bioengineering and Bioinformatics, Lomonosov Moscow State University, 119991 Moscow, Russia;; ^e^Mass Spectrometry Facility, Department of Chemistry, Philipps University Marburg, 35043 Marburg, Germany

**Keywords:** coronavirus, nidovirus, nucleotidyltransferase, RNA polymerase, NiRAN

## Abstract

RNA-dependent RNA polymerases (RdRps) of the *Nidovirales* (*Coronaviridae*, *Arteriviridae*, and 12 other families) are linked to an amino-terminal (N-terminal) domain, called NiRAN, in a nonstructural protein (nsp) that is released from polyprotein 1ab by the viral main protease (M^pro^). Previously, self-GMPylation/UMPylation activities were reported for an arterivirus NiRAN-RdRp nsp and suggested to generate a transient state primed for transferring nucleoside monophosphate (NMP) to (currently unknown) viral and/or cellular biopolymers. Here, we show that the coronavirus (human coronavirus [HCoV]-229E and severe acute respiratory syndrome coronavirus 2) nsp12 (NiRAN-RdRp) has Mn^2+^-dependent NMPylation activity that catalyzes the transfer of a single NMP to the cognate nsp9 by forming a phosphoramidate bond with the primary amine at the nsp9 N terminus (N3825) following M^pro^-mediated proteolytic release of nsp9 from N-terminally flanking nsps. Uridine triphosphate was the preferred nucleotide in this reaction, but also adenosine triphosphate, guanosine triphosphate, and cytidine triphosphate were suitable cosubstrates. Mutational studies using recombinant coronavirus nsp9 and nsp12 proteins and genetically engineered HCoV-229E mutants identified residues essential for NiRAN-mediated nsp9 NMPylation and virus replication in cell culture. The data corroborate predictions on NiRAN active-site residues and establish an essential role for the nsp9 N3826 residue in both nsp9 NMPylation in vitro and virus replication. This residue is part of a conserved N-terminal NNE tripeptide sequence and shown to be the only invariant residue in nsp9 and its homologs in viruses of the family *Coronaviridae*. The study provides a solid basis for functional studies of other nidovirus NMPylation activities and suggests a possible target for antiviral drug development.

## Significance

We report an intersubunit interaction within the coronavirus replication–transcription complex that is critical for replication and evolutionarily conserved. We provide evidence that the nsp12-associated NiRAN domain has nucleoside monophosphate (NMP) transferase activity in trans and identified nsp9, an RNA-binding protein, as its target. NiRAN catalyzes the covalent attachment of an NMP moiety to the conserved nsp9 amino terminus in a reaction dependent on Mn^2+^ ions and an adjacent conserved Asn residue. NiRAN activity and nsp9 NMPylation were found to be essential for coronavirus replication. The data lead us to connect this activity of a nidovirus enzymatic marker with previous observations within a functionally and evolutionarily coherent hypothesis on the initiation of RNA synthesis in a class of RNA viruses.

Positive-strand RNA viruses of the order *Nidovirales* infect diverse vertebrates and invertebrates (1, 2). The order currently comprises 14 families (3), of which the *Coronaviridae* have been studied extensively in the past 2 decades when three zoonotic coronaviruses emerged from animal reservoirs and caused major outbreaks of severe respiratory infections in humans, including the ongoing pandemic caused by severe acute respiratory syndrome coronavirus 2 (SARS-CoV-2) (4–7). Nidoviruses share a common genome organization, with subunits of the membrane-bound replication–transcription complex (RTC) encoded in the 5′-terminal two-thirds and the major structural subunits of virus particles, along with a few “accessory” proteins, encoded in the 3′-terminal one-third of the genome (1). Except for a single family of planarian viruses (*Mononiviridae*) (8), all nidoviruses encode RTC subunits in two large open reading frames (ORFs), ORF1a and ORF1b, which are translated from the genomic RNA. ORF1a encodes polyprotein (pp) 1a, and ORF1a and ORF1b encode jointly pp1ab. Both pp1a and pp1ab are proteolytically processed to multiple nonstructural proteins (nsps) with the universal involvement of the ORF1a-encoded main protease (M^pro^), also known as 3CL^pro^ due to its homology to the 3C^pro^ of picornaviruses (9). These nsps are thought to assemble into large and dynamic RTCs that catalyze the synthesis of genomic RNA (replication) and a set of subgenomic RNAs (transcription) that are used for the coordinated expression of ORFs located downstream of ORF1b (10–12).

The core RTC includes the RNA-dependent RNA polymerase (RdRp) (13), a superfamily 1 helicase (HEL1) (14, 15) and several RNA-processing enzymes, which are predominantly encoded in ORF1b and comprise nsp12-nsp16 in the *Coronaviridae* and nsp9-nsp12 in the *Arteriviridae* (reviewed in refs. 10–12). RdRp and HEL1 represent two (out of five) nidovirus-wide conserved domains and have homologs in other RNA viruses. The core replicative enzymes are believed to be assisted by other subunits, including several small nsps that are released from the carboxyl-terminal (C-terminal) region of pp1a, downstream of the M^pro^ (coronavirus nsp5 and arterivirus nsp4, respectively). They have limited family-specific conservation and diverse activities (reviewed in refs. 10–12).

Relatively recently, a domain with a distinctive sequence motif signature was identified amino-terminally (N-terminally) adjacent to the RdRp in all nidoviruses but no other RNA virus (16). Based on its location and nucleotidyltransferase (nucleoside monophosphate [NMP] transferase) activity, the domain was named NiRAN (nidovirus RdRp-associated nucleotidyltransferase). The two-domain combination of NiRAN-RdRp makes up nsp12 in the family *Coronaviridae* and nsp9 in the family *Arteriviridae*, and also in other nidovirus families, NiRAN-RdRp is expected to be released as a separate nsp from the viral polyprotein(s). In coronaviruses, the NiRAN domain comprises ∼250 residues and is linked to the C-terminal RdRp domain via a linker region (16–19). In equine arteritis virus (EAV) (family *Arteriviridae*), a recombinant nsp9 was shown to have Mn^2+^ ion-dependent (self-)UMPylation and GMPylation activities that depend on residues in three sequence motifs conserved in nidoviruses, A_N_, B_N_, and C_N_ (where N stands for NiRAN) (16). The motifs are N-terminally flanked by a less conserved motif preA_N_. Some of these residues are also conserved in distantly related protein kinases in which they have been shown to be involved in nucleoside triphosphate (NTP) binding and catalytic activity (20, 21). Consistent with this observation, several key active-site residues in the pseudokinase SelO from *Pseudomonas syringae* could be superimposed with conserved coronavirus NiRAN residues in a recently published cryoelectron microscopic structure of a SARS-CoV-2 nsp7/8/12/13 supercomplex assembled from recombinant proteins (17). The documented (self-)U/GMPylation was speculated to generate a transient state that is primed for transferring NMP to (currently unknown) substrates (16), and the structural similarities of NiRAN to protein kinases (17, 19) lent support to the hypothesis that NiRAN modifies other proteins.

A host of characteristics, including its exclusive and singular phyletic association with nidoviruses and its genetic segregation with the RdRp, makes NiRAN a plausible key regulator enzyme of nidoviruses that is central to their emergence and identity. Previously, three possible functions implicating NiRAN in the regulation of either genome/subgenome translation or replication/transcription were invoked, with each having its pros and cons when considered against the scarce and incomplete data available at the time (16). In this study, we aimed at gaining insight into this enigmatic domain by combining biochemical and reverse-genetics studies of coronaviruses representing two genera and putting our findings in an evolutionary context of the natural variation of the *Coronaviridae* family. We report a major advancement in our understanding of the NiRAN by identifying its natural target in the RTC, which (among the three available hypotheses) favors a role of this domain in the initiation of nidovirus RNA synthesis. The study also opens a possibility for additional roles of NiRAN at the virus–host interface.

## Results

### Self-NMPylation Activity of the Coronavirus NiRAN Domain.

**Fig. 1.**
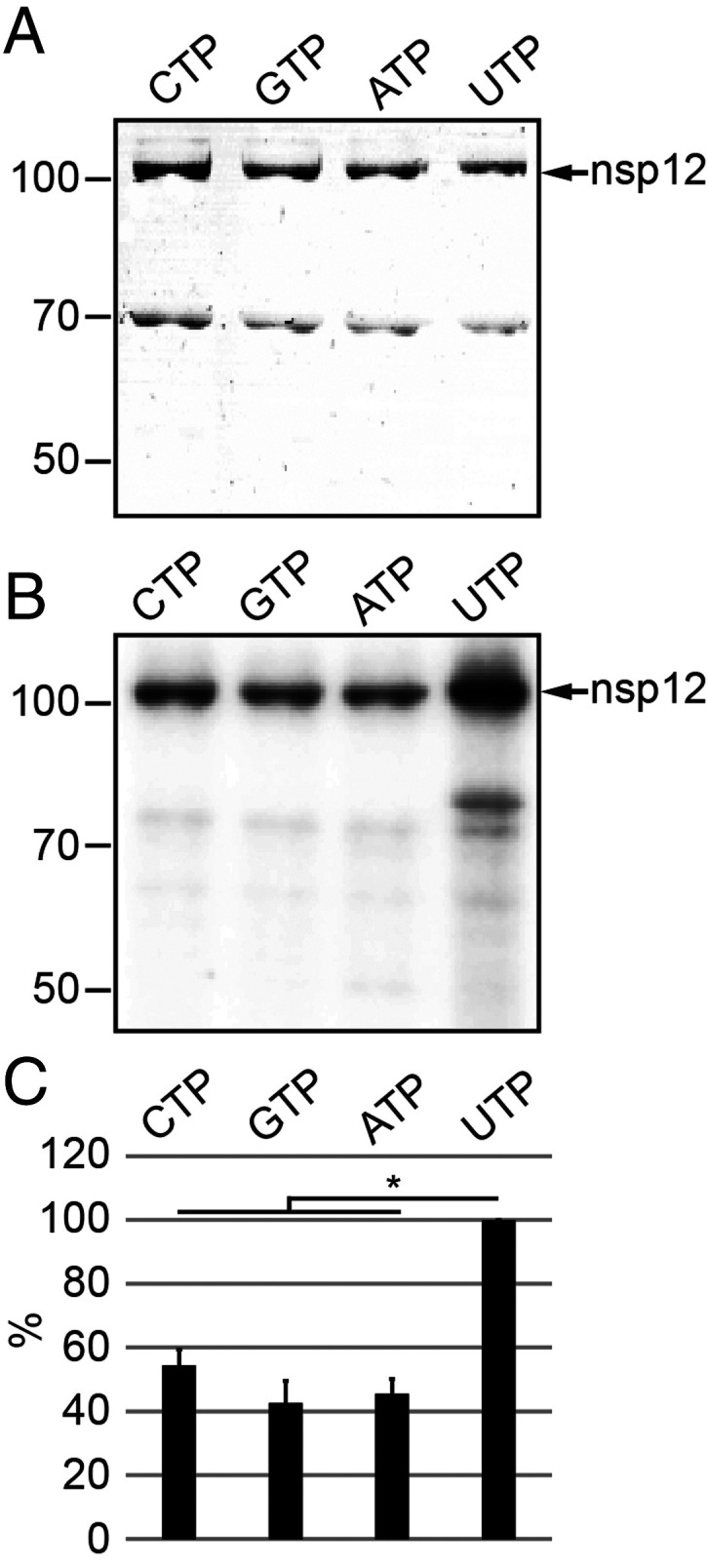
Self-NMPylation activity of HCoV-229E nsp12. (*A*) HCoV-229E nsp12-His_6_ (106 kDa) was incubated for 30 min with the indicated [α-^32^P]NTPs in the presence of 6 mM MnCl_2_ (for details, see *Materials and Methods*). Reaction products were separated by SDS-PAGE and stained with Coomassie brilliant blue. (*B*) Radiolabeled proteins were visualized by phosphorimaging. Positions of nsp12-His_6_ and protein molecular mass markers (in kilodaltons) are indicated in *A* and *B*. (*C*) Radioactive signal intensities (mean values ± SEM) were determined from three independent experiments. *P ≤ 0.05. Signal intensities (in percentages) are given in relation to UTP.

To characterize the enzymatic properties of coronavirus nsp12-associated NiRAN domains, we produced a recombinant form of human coronavirus 229E (HCoV-229E) nsp12 with a C-terminal His_6_ tag in *Escherichia* *coli* and incubated the protein with [α^32^-P]NTPs in the presence of MnCl_2_ as described in *Materials and Methods*. Analysis of the reaction products revealed the presence of radiolabeled proteins that comigrated with nsp12 (106 kDa), suggesting that coronavirus nsp12 catalyzes the formation of covalent protein-NMP adducts, preferentially with uridine monophosphate (UMP) (Fig. 1 *A *and* B*). Quantitative analyses revealed a 2- to 3-fold increased signal intensity for UMP incorporation compared to other nucleotides (Fig. 1*C*). The data are consistent with the predicted NMP transferase activity for coronavirus NiRAN domains (16) but indicate different nucleotide preferences for coronavirus and arterivirus NiRAN domains.

### NiRAN-Mediated NMPylation of nsp9.

**Fig. 2.**
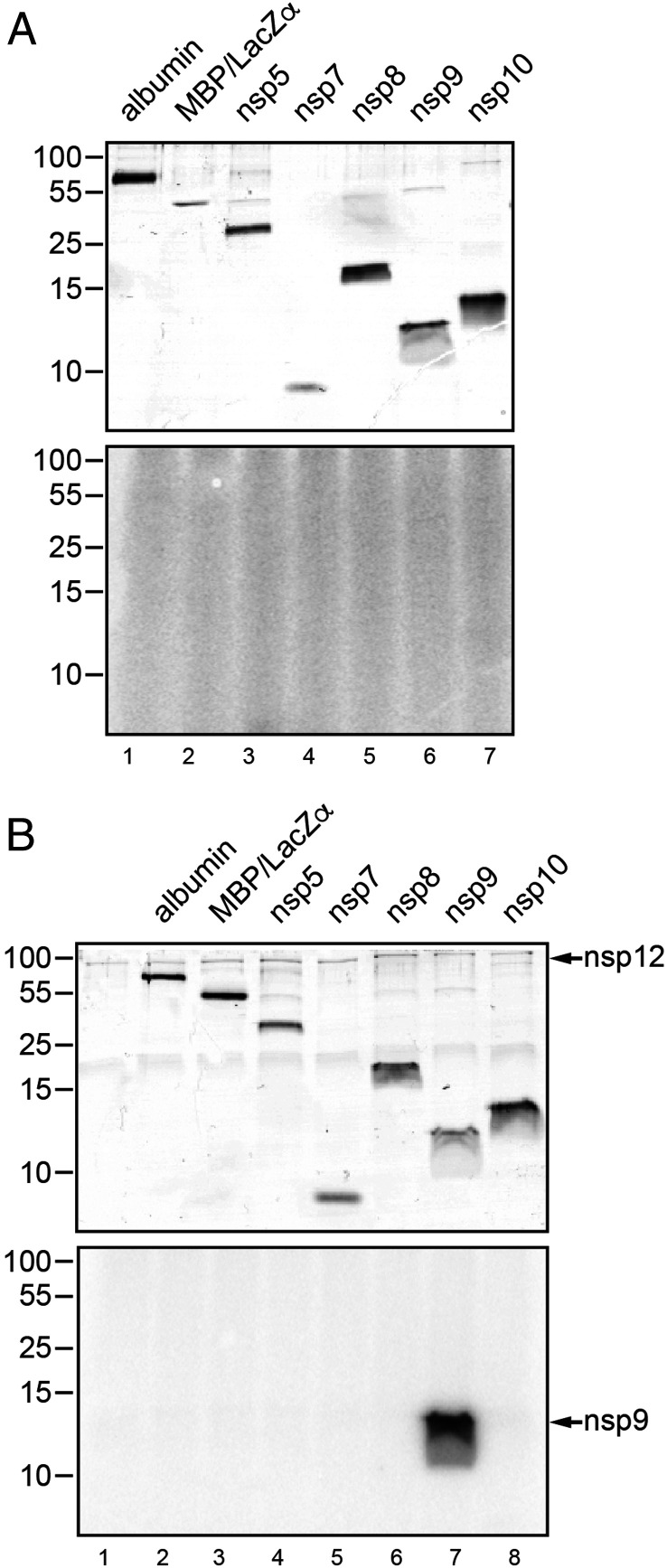
HCoV-229E nsp12-mediated UMPylation of nsp9. HCoV-229E nsp12-His_6_–mediated protein UMPylation activity was assessed using a range of protein substrates, including bovine serum albumin, MBP-lacZα, and a range of C-terminally His_6_-tagged HCoV-229E nsps encoded by ORF1a. The proteins were incubated for 10 min with [α-^32^P]UTP in the absence (*A*) or presence (*B*) of nsp12, as described in *Materials and Methods*. In the *Top* of *A* and *B*, the Coomassie brilliant blue-stained SDS-polyacrylamide gels are shown, and, in the *Bottom* of *A* and *B*, the corresponding autoradiograms are shown. Positions of protein molecular mass markers (in kilodaltons) are given to the left. Also indicated is the position of nsp12-His_6_ (*B*, *Top*) and the radioactive signal observed upon incubation of nsp12-His_6_ with nsp9-His_6_ (*B*, lane 7), the latter indicating nsp12-His_6_–mediated transfer of [α-^32^P]UMP to nsp9-His_6_ (12.9 kDa), which was not observed for other proteins tested.

**Fig. 3.**
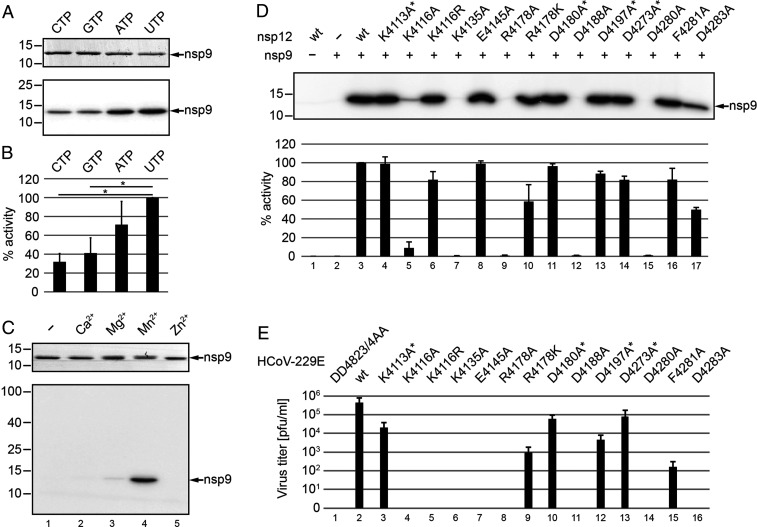
Biochemical and virological characterization of HCoV-229E NiRAN-mediated nsp9 NMPylation. (*A* and *B*) Role of nucleotide cosubstrate used in the reaction. Nsp12-His_6_ and nsp9-His_6_ were mixed and incubated in the presence of different [α-^32^P]NTPs in the standard NMPylation assay. (*A*, *Top*) The Coomassie-stained nsp9-His_6_ separated by SDS-PAGE. (*A*, *Bottom*) An autoradiogram of the same area of the gel. (*B*) Relative activities (mean values ± SEM) in the presence of the indicated nucleotide cofactors were determined from three independent experiments. *P ≤ 0.05. (*C*) Role of metal ions. Shown is a standard NMPylation assay in the presence of [α-^32^P]UTP and different metal ions, each at 1 mM. In *C*, *Top*, the Coomassie-stained nsp9-His_6_ is shown, and, in *C*, *Bottom*, the corresponding autoradiogram is shown. Sizes of marker proteins (in kilodaltons) are indicated to the left in *A* and *C*. (*D*) Mutant forms of HCoV-229E nsp12-His_6_ carrying the indicated amino acid substitutions were incubated with nsp9-His_6_ in the presence of [α-^32^P]UTP, as described in *Materials and Methods*. Radiolabeled nsp9-His_6_ produced in the NMPylation reaction was detected by phosphorimaging (*D*, *Top*), and relative activities compared to the wild-type (wt) protein are presented in *D*, *Bottom* as mean values (±SEM) determined from three independent experiments. Asterisks indicate substitutions of nonconserved residues. (*E*) Virus titers in p1 cell culture supernatants obtained at 24 h p.i. were determined by plaque assay. Codon substitutions in the NiRAN domain of the engineered HCoV-229E mutants are indicated (residue numbering is according to their position in pp1ab). The replication-deficient RdRp active-site mutant nsp12_DD4823/4AA was used as control.

Although NiRAN-associated enzymatic activities were shown to be essential for EAV and SARS-CoV replication in cell culture (16), specific NiRAN functions and potential targets have not been identified. The recently reported structural similarities of NiRAN to a family of proteins with a protein kinase-like fold (17, 22) prompted us to test the hypothesis that NiRAN catalyzes the NMPylation of other proteins. We produced a set of potential cognate targets including HCoV-229E ORF1a-encoded nonstructural proteins (nsps 5, 7, 8, 9, 10), each containing a C-terminal His_6_ tag (*SI Appendix*, Table S1), and incubated these proteins with [α^32^-P]uridine triphosphate ([α^32^-P]UTP) in the presence or absence of nsp12. Bovine serum albumin and an MBP-LacZα fusion protein produced in *E. coli* served as controls (Fig. 2*A*, lanes 1 to 7). Analysis of radiolabeled proteins by sodium dodecyl sulphate–polyacrylamide gel electrophoresis (SDS-PAGE) and autoradiography revealed an intense radioactive signal in the reaction containing both nsp12 and nsp9. The position of the signal corresponded to the molecular mass of nsp9, suggesting an nsp12-mediated UMPylation of nsp9 (Fig. 2*B*, lane 7). None of the other test proteins was found to be UMPylated, which led us to conclude that nsp9 is a specific substrate of nsp12. Consistent with the self-NMPylation data shown in Fig. 1, nsp12 was able to transfer all four NMPs to nsp9, albeit with different efficiencies, UMP > adenosine monophosphate (AMP) > guanosine monophosphate (GMP) > cytidine monophosphate (CMP) (Fig. 3 *A* and *B*). Under the conditions used in this assay (shortened reaction and exposure times, reduced concentration of nsp12; *Materials and Methods*), self-NMPylation of nsp12 was not detectable (compare Fig. 2*B*, lane 7, with Fig. 1*B*), demonstrating an efficient (and multiple-round) transfer of UMP from nsp12 to nsp9. The UMP transferase activity required the presence of Mn^2+^ ions, as shown in Fig. 3*C*, while only minimal UMP transferase activity was observed in the presence of Mg^2+^ and no activity in the presence of the two other divalent cations tested. Similar data were obtained in NMPylation assays containing cytidine triphosphate (CTP), guanosine triphosphate (GTP), and adenosine triphosphate (ATP), respectively (*SI Appendix*, Fig. S1).

### Identification of NiRAN Residues That Are Critically Involved in nsp9 NMPylation and Virus Reproduction.

To obtain more insight into the NiRAN active site and identify residues involved in nsp9-specific NMP transferase activity, we performed a mutational analysis in which we substituted conserved residues in the NiRAN A_N_, B_N_, and C_N_ motifs (16) with Ala (*SI Appendix*, Fig. S2). In addition, effects of conservative Arg-to-Lys or Lys-to-Arg substitutions were assessed in two cases. As (negative) controls, residues that were not, or were less, conserved among corona- and other nidovirus NiRAN domains were replaced with Ala. The substitutions K4116A (in motif preA_N_), K4135A (A_N_), R4178A (B_N_), D4188A (motif B_N_), and D4280A (C_N_) profoundly reduced or even abolished nsp9 NMPylation by nsp12, while proteins with conservative replacements (R4178K, K4116R) retained ∼60 and 80% of their activities, suggesting somewhat relaxed constraints for the respective side chains that are physicochemically sensible (Fig. 3*D*). Substitutions of several other conserved residues, E4145A, D4273A, F4281A, and D4283A, were much less detrimental, with nsp9 UMPylation being only moderately reduced. Similar results were obtained in nsp9 NMPylation reactions containing other NTPs (Fig. 3*D* and *SI Appendix*, Fig. S3), confirming that the effects observed for specific amino acid substitutions were independent of the type of nucleotide cosubstrate used. Next, we tested possible effects of these nsp12 substitutions on coronavirus replication in cell culture. To this end, we transcribed 5′-capped genome-length HCoV-229E RNAs carrying the respective nsp12 substitutions using appropriate genetically engineered complementary DNA (cDNA) templates cloned in recombinant vaccinia viruses (23, 24) and transfected these RNAs into Huh-7 cells. Titration of infectious virus progeny produced in these cells revealed that a majority of the HCoV-229E NiRAN mutants were not viable (Fig. 3*E*). The set of nonviable virus mutants included replacements shown to abolish or profoundly reduce NMP transferase activity in vitro (K4116A, K4135A, R4178A, D4188A, D4280A, D4283A) but also two other replacements (K4116R, E4145A) that retained ≥80% of their in vitro NMPylation activity, indicating an involvement of additional constraints. Likewise, two other mutations causing a moderate reduction of NiRAN in vitro NMPylation activity (R4178K, F4281A) gave rise to viable viruses that, however, replicated to significantly reduced titers. Consistent with the in vitro activity data shown in Fig. 3*D*, replacements of four other residues that are not conserved among corona- and/or other nidoviruses (K4113A, D4180A, D4197A, D4273A) (8, 16) resulted in viable virus progeny, albeit with moderately reduced titers compared to the wild-type virus (Fig. 3*E*).

To investigate whether the NiRAN-mediated NMP transferase activity depends on an active RdRp domain, two conserved Asp residues in the RdRp motif C that are involved in divalent metal ion coordination (11) were replaced with Ala. The resulting protein, nsp12_DD4823/4AA, retained its nsp9 NMPylation activity, indicating that the polymerase activity is not required for nsp12-mediated nsp9 NMPylation activity in vitro (*SI Appendix*, Fig. S4).

### NiRAN Catalyzes the Covalent Attachment of a Single NMP Moiety to the nsp9 N Terminus.

**Fig. 4.**
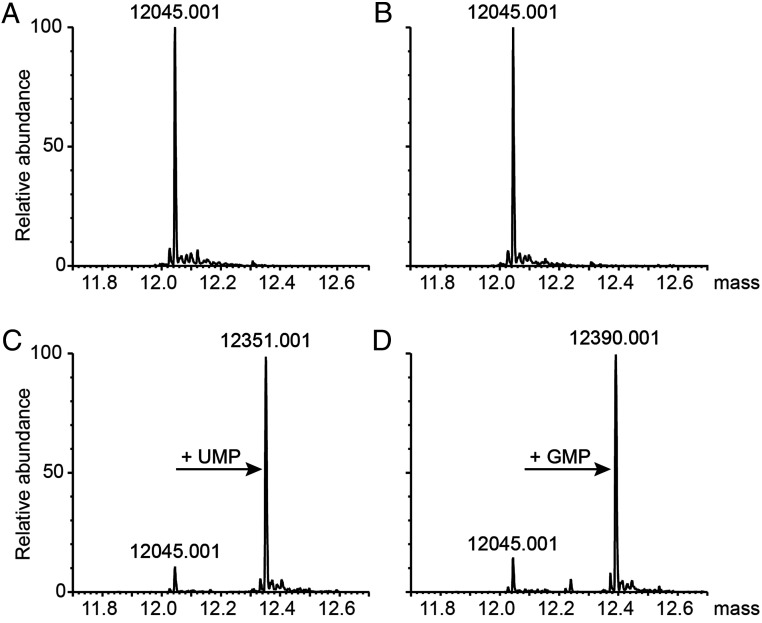
Mono-NMPylation of nsp9 in the presence of nsp12 and UTP or GTP. Shown are deconvoluted intact protein mass spectra of HCoV-229E nsp9 (*SI Appendix*, Table S1) (*A*–*D*). (*A*) nsp9 alone, (*B*) nsp9 + nsp12-His_6_, (*C*) nsp9 + nsp12-His_6_ in the presence of UTP, (*D*) nsp9 + nsp12-His_6_ in the presence of GTP.

Having established an nsp9-specific NMP transferase activity for nsp12, we sought to characterize NMP-nsp9 adducts by mass spectrometry (MS). The spectrum of the intact protein mass of recombinant HCoV-229E nsp9 displayed a peak at 12,045 Da (Fig. 4*A*). The addition of nsp12 did not change the mass of nsp9, indicating that nsp12 and nsp9 do not form stable complexes under the (denaturing) conditions used (Fig. 4*A*). Mass measurements of reactions containing nsp9 and nsp12 in the presence of UTP and GTP, respectively, revealed a protein mass shift by 306 Da for UTP and 345 Da for GTP, indicating that a single UMP or GMP per nsp9 molecule was bound (Fig. 4 *C* and *D*). Presumably, the energy required for NiRAN-mediated nsp9 NMPylation was derived from NTP hydrolysis and pyrophosphate release. Although a 10-fold molar excess of nsp9 (target) over nsp12 (enzyme) was used in this reaction, a nearly complete NMPylation of nsp9 was observed, indicating that the interaction between nsp12 and nsp9 is transient and nsp12 is able to NMPylate multiple nsp9 molecules in vitro.

To identify the nsp9 residue that is UMPylated by nsp12, nsp9-UMP was cleaved using trypsin and the resulting peptides were separated by nano–high-performance liquid chromatography (HPLC) and analyzed online by tandem MS (MS/MS). Data analysis using the Byonic software package (Protein Metrics) indicated the UMPylation of the N-terminal amino acid. This was confirmed manually. The tandem mass spectrum of the precursor peptide [UMP]NNEIMPGK (*SI Appendix*, Fig. S5*A*) revealed a fragment at 421 *m*/*z*, indicating that UMP was bound to residue 1 of nsp9.

At the nsp9 N terminus, Asn is conserved among members of the *Orthocoronavirinae* (*SI Appendix*, Fig. S6). Although we considered the nitrogen atom of the N-terminal primary amine to be the most likely acceptor for UMP, we decided to obtain additional evidence for NMP binding at the N terminus. To this end, HPLC-purified non-NMPylated and NMPylated N-terminal peptides of nsp9 were derivatized in the presence of acetone and sodium cyanoborohydride. Under these conditions, only free primary amines can be modified with a propyl group (25). The N-terminal nsp9-derived peptide with the sequence NNEIMPGK contains two primary amines, one at the N terminus of Asn and one at the side chain of the C-terminal Lys. Consequently, propyl groups could be introduced at both termini. The extracted ion chromatograms of the non-NMPylated peptide are shown in the *SI Appendix*, Fig. S5*B*. As expected, N- and C-terminally (mono)propylated (*SI Appendix*, Fig. S5*B*, top lane) and dipropylated peptides (*SI Appendix*, Fig. S5*B*, bottom lane) could be identified. This pattern changed with the use of NMPylated N-terminal peptide of nsp9. In this case, only a C-terminally propylated peptide but no N-terminally propylated peptide and no dipropylated peptide could be identified (*SI Appendix*, Fig. S5*C*), suggesting that the UMP had been transferred to the N-terminal primary amine, thereby blocking this group for modification.

### A Free N Terminus and the Second Asn Residue of nsp9 Are Essential for nsp9 NMPylation.

**Fig. 5.**
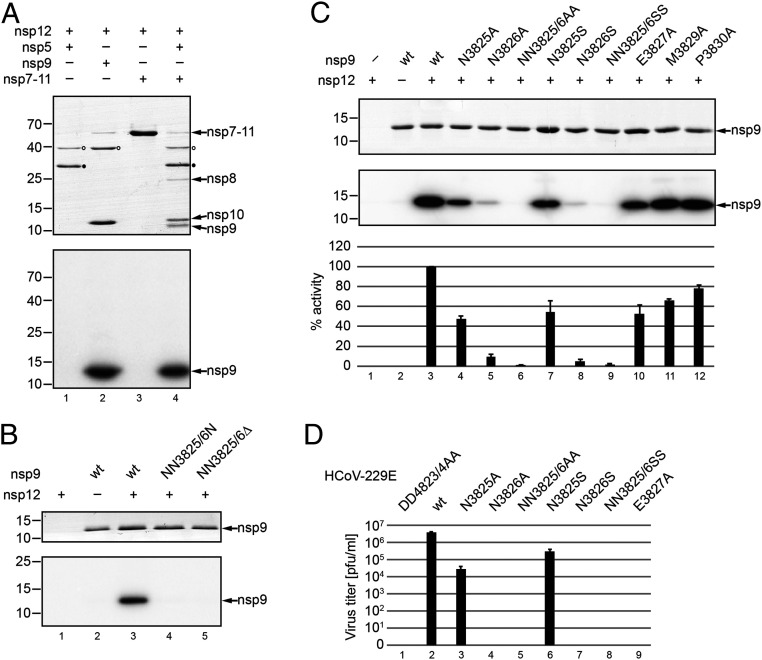
Roles of proteolytic processing and N-terminal residues of nsp9 in nsp12-mediated UMPylation. (*A*) Requirement of a free nsp9 N terminus for nsp9 UMPylation. Nsp7-11-His_6_ was preincubated at 30 °C in the presence or absence of recombinant M^pro^ (nsp5-His_6_) in NMPylation assay buffer containing UTP. After 3 h, the NMPylation assay was started by the addition of nsp12-His_6_, as described in *Materials and Methods*. Reactions containing nsp5-His_6_ (lane 1) and nsp9-His_6_ (lane 2) were used as controls. After 10 min, the reactions were terminated and the reaction mixtures separated by SDS-PAGE. Proteins were stained with Coomassie brilliant blue (*A*, *Top*). Nsp7-11-His_6_ precursor and processing products resulting from nsp5-His_6_–mediated cleavage are indicated to the right. Note that (due to their small size) nsp7 and nsp11-His_6_ are not detectable in this gel and reactions supplemented with nsp5-His_6_ (lanes 1 and 4; position of nsp5-His_6_ is indicated by a closed circle) or nsp9-His_6_ (lane 2) contain small amounts of MBP (indicated by open circles) as a residual impurity resulting from their expression as MBP fusion proteins (*SI Appendix*, Table S1). (*B*) Nsp9-His_6_ variants lacking one or two N-terminal Asn residues (residue numbering is according to the position in pp1a/pp1ab) were purified and incubated with nsp12-His_6_ and [α-^32^P]UTP. *B*, *Top* shows the Coomassie-stained SDS-PAGE and *B*, *Bottom* shows the corresponding autoradiogram. Positions of molecular weight markers (in kilodaltons) are indicated to the left. (*C*) Conserved residues at the N terminus of HCoV-229E nsp9-His_6_ were replaced with Ala or Ser, and equal amounts of protein were used in nsp12-His_6_–mediated UMPylation reactions. Reaction products were separated by SDS-PAGE and stained with Coomassie brilliant blue (*C*, *Top*), and radiolabeled nsp9-His_6_ was detected by phosphorimaging (*C*, *Middle*). Relative NMPylation activities (mean values ± SEM) were calculated from three independent experiments using the wild-type (wt) protein as a reference (set to 100%). (*D*) Virus titers in p1 cell culture supernatants of Huh-7 cells infected with HCoV-229E wild type and mutants carrying the indicated amino acid replacements in nsp9 were determined by plaque assay. The replication-deficient RdRp motif C double mutant, DD4823/4AA, was used as negative control.

Next, we substituted (with Ala or Ser) or deleted conserved residues at the nsp9 N terminus to define target-specific constraints. Based on our MS data suggesting that NiRAN forms an nsp9-NMP adduct with the primary amine of the nsp9 N-terminal residue, we hypothesized that nsp9 NMPylation requires proteolytic cleavage of the nsp8|9 peptide bond by the viral main protease (M^pro^, nsp5) to release the nsp9 N terminus from its polyprotein precursor. To test this hypothesis, we produced an nsp9-containing precursor protein, nsp7-11, in *E. coli* and performed a standard NMPylation assay in the presence of [α-^32^P]UTP (*Materials and Methods*). As shown in Fig. 5*A* (lane 3), the uncleaved nsp7-11 precursor was not radiolabeled by nsp12. In contrast, if nsp7-11 was cleaved with recombinant nsp5 to release nsp9 (and other nsps) from this precursor, a radiolabeled protein comigrating with nsp9 was detected, corroborating our conclusion that NiRAN forms selectively a covalent nsp9-NMP adduct with the N-terminal primary amine of the N-terminal Asn (position 3825 in pp1a/pp1ab). This conclusion was also supported by experiments using nsp9 constructs containing one or two additional residues at the N terminus. In both cases, NiRAN-mediated UMPylation of nsp9 was abolished (*SI Appendix*, Fig. S7). Next, we produced proteins in which one or two Asn residues were deleted from the nsp9 N-terminal 3825-NNEIMPGK-3832 peptide sequence. In both cases, nsp9 UMPylation was completely blocked (Fig. 5*B*), providing additional evidence that the authentic nsp9 N terminus acts as NMP acceptor.

The nsp9 N terminus (especially positions 1, 2, 3, and 6) is well conserved among members of the subfamily *Orthocoronavirinae* (*SI Appendix*, Fig. S6). To investigate possible roles of these residues in nsp12-mediated nsp9 NMPylation, the two consecutive Asn residues at the nsp9 N terminus were substituted with Ala or Ser (alone or in combination). Substitution of N3825 with either Ala or Ser resulted in a greater than twofold reduction of nsp12-mediated UMPylation compared to wild-type nsp9 (Fig. 5*C*). Consistent with our conclusion that NMPylation occurs at the N-terminal primary amine rather than the side chain of the N-terminal residue, we observed a significant residual NMPylation for the N3825A and N3825S replacements. Interestingly, nsp9 UMPylation was much more strongly decreased (by more than 10-fold) if the second Asn was replaced with either Ala or Ser, while Ala replacements at positions 3, 4, and 6 had only moderate effects on nsp9 UMPylation (Fig. 5*C*). Similar results were obtained using ATP, CTP, or GTP (*SI Appendix*, Fig. S8). Collectively, these data suggest a key role for N2826 (position 2 in nsp9) in nsp9 NMPylation.

### The Second Asn Is the Only Invariant Residue in nsp9 Homologs of the Family *Coronaviridae*.

To obtain additional evidence for the functional relevance of the nsp9 N terminus for NMPylation, we did a multiple-sequence alignment (MSA) of nsp9 sequences of the family *Coronaviridae* (varying between 104 to 113 residues) (*SI Appendix*, Fig. S6). In total, only 8 residues were found to be invariant in (all of) the 47 (known and putative) species of 5 genera of the subfamily *Orthocoronavirinae* infecting diverse mammalian, avian, and reptile hosts. The most extensive variations, including deletions and insertions, were observed in loops between secondary structure elements of nsp9, as determined in previous structural studies (26–28). Five invariant residues were found in β-strands and the α-helix in the C-terminal part of nsp9, and three invariant residues constitute the NNE motif at the very N terminus of nsp9. The second Asn of this motif was revealed to be the only invariant residue that is also shared by the putative nsp9 of the distantly related frog coronavirus representing the species *Microhyla letovirus 1* in the genus *Alphaletovirus*, subfamily *Letovirinae*. The conservation of residues in secondary structure elements of nsp9 can be rationalized by either structural considerations to maintain the fold or the known RNA-binding properties of this protein. However, this reasoning seems poorly applicable to the NNE conservation and, prior to this study, left the nature of the constraints that limit the variation of this tripeptide sequence totally obscure.

### The Restricted Natural Variation of the N-Terminal Tripeptide of nsp9 Is Significant for HCoV-229E Replication in Cell Culture.

To determine the importance of nsp9-NMPylation and NNE conservation in coronavirus replication, we produced HCoV-229E mutants carrying single or double replacements of nsp9 N-terminal residues shown to be detrimental for nsp9 NMPylation in vitro. Before we embarked on this, we sought to answer the question of whether these substitutions (near the nsp8|9 cleavage site) might affect the proteolytic processing of the C-terminal pp1a region. A set of nsp7-11 polyprotein constructs containing the respective substitutions at the nsp9 N terminus were produced in *E. coli* and cleaved with recombinant M^pro^. Proteolytic cleavage at four sites, including the nsp9 flanking sites, was not evidently affected by any of the substitutions introduced (*SI Appendix*, Fig. S9), excluding structural changes in these proteins that interfere with M^pro^-mediated cleavage at the nsp8|9 (or other) site(s).

Transfection of Huh-7 cells with genome-length HCoV-229E RNAs encoding Ala or Ser substitutions in the conserved NNE tripeptide (N3825, N3826, and E3827) at the nsp9 N terminus revealed that most mutations were lethal. We were able to rescue viruses with a Ser or Ala substitution of the N-terminal Asn (N2835A or N2835S) but failed to recover viruses with other single and double mutations in the NNE sequence (N3826A, N3826S, NN3825/6AA, NN3825/6SS, E3827A) (Fig. 5*D*).

These results suggest that coronavirus replication in tissue culture is under (the same or similar) constraints that limit the natural variation of the nsp9 NMPylation site in vivo, supporting a critical role of this reaction in the coronavirus life cycle.

### SARS-CoV-2 nsp12-Mediated NMPylation of nsp9.

**Fig. 6.**
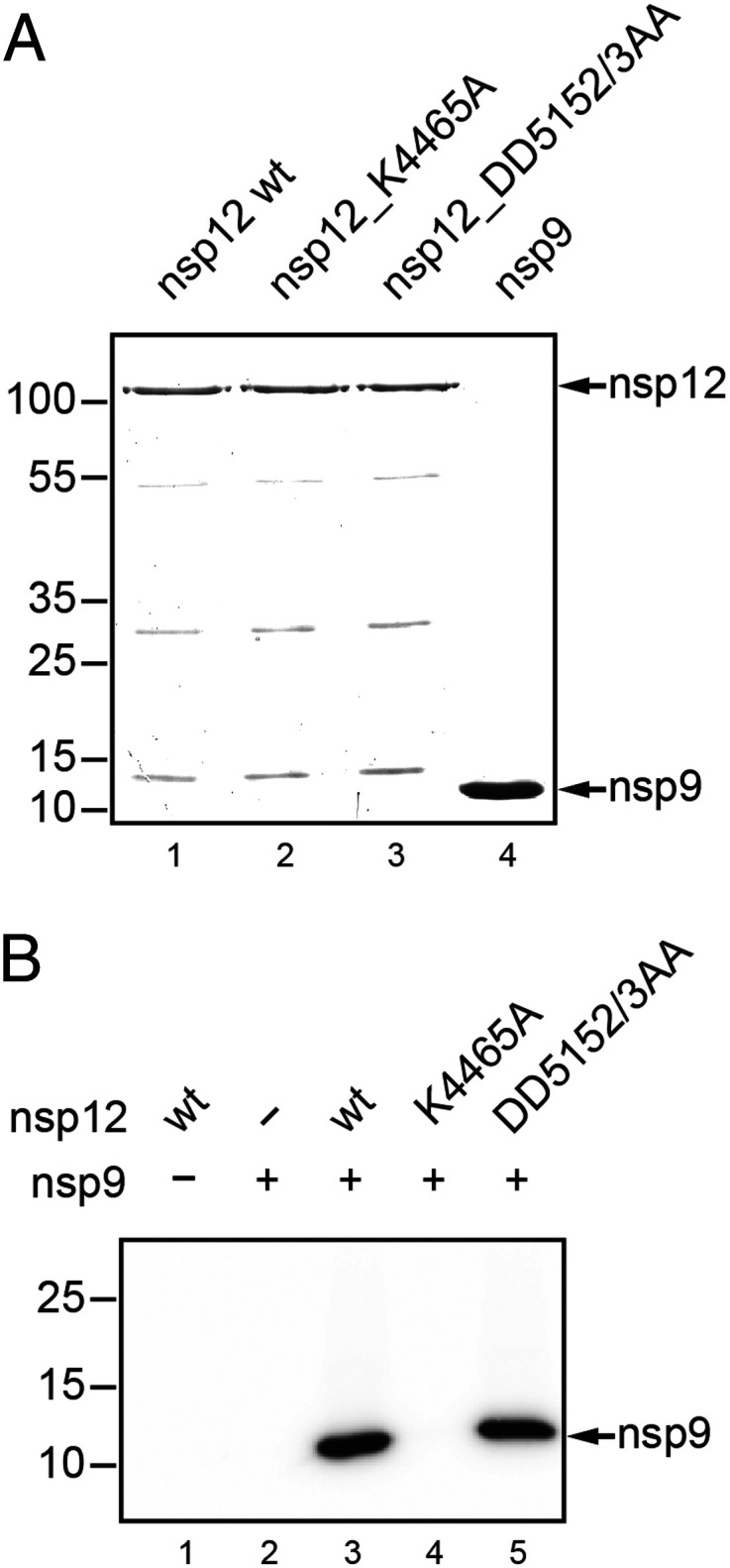
SARS-CoV-2 nsp12-mediated NMPylation of nsp9. (*A*) Coomassie-stained SDS-polyacrylamide gel showing the recombinant proteins used in the NMPylation assay. As controls, mutant proteins with active-site replacements in the NiRAN domain (K4465A) and the RdRp domain (DD5152/3AA) of SARS-CoV-2 nsp12 were used. Residue numbering is according to the position in pp1ab. (*B*) Autoradiogram of an UMPylation assay using nsp9-His_6_ and [α-^32^P]UTP as substrates for nsp12-His_6_ (wild type [wt] and mutants). Molecular masses (in kilodaltons) of marker proteins are indicated to the left.

In a final set of experiments, we produced C-terminally His_6_-tagged forms of SARS-CoV-2 nsp12 and nsp9 in *E. coli*, along with two mutant forms of nsp12 in which an active-site residue in the NiRAN and RdRp domain, respectively, was substituted with Ala (Fig. 6*A* and *SI Appendix*, Table S2). K4465 in SARS-CoV-2 nsp12 corresponds to K4135 in HCoV-229E (*SI Appendix*, Fig. S2), which was shown to be essential for NiRAN activity and HCoV-229E replication (Fig. 3 *D* and *E*). The residue also corresponds to the arterivirus EAV nsp9 K94 residue that was shown previously to be essential for NiRAN self-UMPylation/self-GMPylation (16). As shown in Fig. 6*B*, SARS-CoV-2 nsp12 has UMP transferase activity using nsp9 as a substrate, while the nsp12_K4465A active-site mutant was inactive. The double substitution in the SDD signature sequence of the RdRp motif C did not affect UMP transferase activity (Fig. 6*B*), suggesting that the RdRp activity has no direct role in nsp9 UMPylation. Similar data were obtained using CTP, GTP, and ATP (*SI Appendix*, Fig. S10). Collectively, these data show that NiRAN-mediated NMPylation of nsp9 is a conserved activity in coronaviruses representing different genera of the subfamily *Orthocoronavirinae*.

## Discussion

NiRAN domains are universally conserved in the *Nidovirales* (16), suggesting that they catalyze an essential enzymatic reaction in nidovirus replication. In this study, we were able to show that coronavirus NiRAN domains transfer NMP (generated from NTP) to nsp9, an enigmatic RNA-binding protein involved in viral replication (26–29), identifying it as a natural target and partner in the coronavirus RTC.

### Coronavirus NiRAN-Mediated NMP Transferase Activity Employs an Active Site Related to SelO-Like Protein Kinases and Is Essential for Viral Replication.

NiRAN domains share three sequence motifs (A_N_, B_N_, and C_N_) containing a very small number of residues that are conserved among all families in the monophyletic but highly diverged *Nidovirales* order (8, 16). Recent studies suggest that they are structurally related to a largely uncharacterized family of protein kinase-like proteins that were originally referred to as the SelO family (17, 19, 22, 30, 31). SelO-related proteins have a kinase fold but lack several active-site residues conserved in canonical kinases (22, 32). Based on the inverted orientation of an ATP molecule bound in the active site and stabilized by specific interactions, SelO was hypothesized and subsequently confirmed to transfer AMP (rather than phosphate) to protein substrates (22), while another bacterial SelO-like protein, YdiU, was shown very recently to catalyze the covalent attachment of UMP to Tyr and His residues of diverse protein substrates (33).

To corroborate and extend predictions made on the putative active-site residues of coronavirus NiRAN domains, we performed a mutational analysis of a coronavirus nsp12 using biochemical and reverse-genetics approaches (Fig. 3 *D* and *E* and *SI Appendix*, Fig. S3 and Tables S1–S4). The data revealed that substitutions of HCoV-229E K4135, R4178, and D4280 with Ala abolished NMP transferase activity in vitro and viral replication in cell culture (Fig. 3*D* and *E* and *SI Appendix*, Fig. S3), supporting their predicted roles in the coordination of the NTP γ-phosphate (K4135, R4178) and active-site metal ion(s) (D4280). The E4145A substitution of a nidovirus-wide conserved Glu predicted to stabilize the position of K4135 (17) was shown to abolish viral replication but, surprisingly, retained activity in the in vitro NMPylation assay (Fig. 3 *D* and *E* and *SI Appendix*, Fig. S3 and Tables S1–S4). Similar observations were made when the corresponding substitution was introduced in the *Salmonella typhimurium* YdiU homolog (E130A) (33). Together, these data support a regulatory rather than catalytic function for this conserved residue.

The replacement of a nidovirus-wide conserved Phe residue (F4281A) in the HCoV-229E NiRAN domain (8) caused a decrease of NMPylation activity in vitro and a major drop in viral replication in cell culture (Fig. 3 *D* and *E* and *SI Appendix*, Fig. S3). The data are compatible with an important regulatory function for this residue, as shown previously for the homologous DFG motif Phe residue that, in canonical protein kinases, is part of the Mg^2+^-binding loop and helps to assemble the “regulatory spine” required for efficient catalytic activity (32, 34). Substitutions of the K4116 residue (in the preA_N_ motif) with Ala and Arg, respectively, abolished viral replication and, as expected, had differential effects on NMP transferase activity in vitro, depending on the amino acid side chain introduced (Fig. 3 *D* and *E* and *SI Appendix*, Fig. S3). The functional data are consistent with structure information suggesting that this residue establishes interactions with an ATP phosphate (17). In NiRAN domains of other nidovirus families, the position of HCoV-229E pp1a/pp1ab K4116 is occupied by Lys, Arg, or His (8), indicating somewhat relaxed functional constraints for this particular residue. The D4188A and D4283A substitutions abolished or strongly reduced enzymatic activity and abolished virus replication (Fig. 3). Both residues are conserved in most, but not all, nidoviruses (8), suggesting important family-specific but probably noncatalytic functions. Ala substitutions of several other Lys and Asp residues (K4113A, D4180A, D4197A, and D4273A) that are not conserved in *Coronaviridae* or other nidovirus families (8) were used as controls. As expected, these substitutions were largely tolerated, with a slight decrease of both enzymatic activity and viral replication in some cases (Fig. 3 and *SI Appendix*, Fig. S3). Overall, the coronavirus mutagenesis data correspond very well with the EAV NiRAN-RdRp self-GMPylation and reverse-genetics data (16), in which important functions for the EAV nsp9 (ortholog of coronavirus nsp12) residues K94 (corresponding to HCoV-229E K4135), R124 (corresponding to R4178), D132 (corresponding to D4188), D165 (corresponding to D4280), and F166 (corresponding to F4281) had been established. In addition, the HCoV-229E mutagenesis data are consistent with and extend the SARS-CoV reverse-genetics data reported previously (16), as illustrated by the remarkably similar phenotypes observed for the corresponding C_N_ motif Phe-to-Ala mutants SARS-CoV_nsp12-F219A and HCoV-229E_F4281A (Fig. 3 *D* and *E* and *SI Appendix*, Fig. S3 and Tables S1–S4).

In contrast to the EAV ortholog (16) with its pronounced preference for UTP and GTP (in the self-NMPylation reaction), our study revealed that coronavirus NiRAN domains (represented by HCoV-229E and SARS-CoV-2) can efficiently transfer all four NMPs, albeit with a slight preference for UMP (Figs. 1 and 3). The relatively low specificity for a particular NTP cosubstrate is in agreement with the recently reported SARS-CoV-2 nsp7/8/12/13 supercomplex structure where ADP-Mg^2+^ was bound in the NiRAN active site without specific interactions being formed to the adenine moiety (17). In our study, the type of nucleotide used in the NMPylation reaction did not differentially affect the activities of the mutant proteins (*SI Appendix*, Fig. S3), indicating that none of those residues is critically involved in the binding to specific nucleobases. The structural basis and potential biological implications of the differential NTP cosubstrate preferences observed for coronavirus and arterivirus NiRAN domains remain to be studied; they may be genuine or due to limitations of the respective studies. At present, it cannot be ruled out that a potential NMPylator activity (compared to the previously characterized self-NMPylation activity) of the arterivirus NiRAN domain has different cosubstrate preferences, also considering that the similarity between arteri- and coronavirus NiRAN domains is at the limits of sequence-based comparisons (16). In contrast to the pseudokinase SelO, which uses Mg^2+^ as a cofactor, coronavirus and arterivirus NiRAN activities are Mn^2+^-dependent (16) (Fig. 3*C* and *SI Appendix*, Fig. S1). The Mn^2+^ dependence and apparent preference for UTP is an unusual feature of protein NMPylators and was only very recently demonstrated for the *S. typhimurium* YdiU protein, which catalyzes the strictly Mn^2+^-dependent UMPylation of protein chaperones to protect cells from stress-induced depletion of the cellular ATP pool (33).

### Identification of nsp9 as a Biologically Relevant Target of the Coronavirus NiRAN Domain.

The recently described structural similarity of coronavirus NiRAN domains with cellular protein kinases (17, 19) provided additional support for NiRAN’s ability to covalently attach NMP to other proteins that we report in this study. We focused our search for possible NiRAN targets on HCoV-229E ORF1a-encoded proteins that are known to assist directly or indirectly ORF1b-encoded replicative enzymes of the RTC (12, 35). Our experiments provided conclusive evidence for an efficient and specific NMPylation of nsp9 (Fig. 2). If the target protein was used at an 8- to 10-fold molar excess over enzyme (nsp12), nsp9 was confirmed to be completely (mono)NMPylated (Fig. 4). We conclude that the interaction of nsp12 with nsp9 is transient and does not result in a stable complex with nsp9 (in the absence of other RTC subunits). This conclusion is supported by protein–protein interaction studies performed for the SARS-CoV proteome (35). MS analyses identified the primary amine of the nsp9 N-terminal residue as the NMPylation site (*SI Appendix*, Fig. S5). The formation of a phosphoramidate bond with the N-terminal amino group distinguishes the NiRAN-mediated NMPylation activity from *P. syringae* SelO-mediated AMPylation reactions catalyzing the formation of *O*-linked AMP-peptide adducts at Ser, Thr, or Tyr residues (22), while the *S. typhimurium* YdiU forms *O*-linked (with Tyr) and *N*-linked (with His) peptide-UMP adducts. The limited information available for SelO family proteins suggests that members of this large protein family have diverged considerably regarding the peptide-NMP adducts formed, an interesting observation that deserves further investigation.

The data obtained in this study led us to hypothesize that NMPylation of nsp9 requires a free N terminus, which, in the context of viral replication, would be provided by M^pro^-mediated proteolytic cleavage of the nsp8|nsp9 processing site in the replicase polyproteins pp1a and pp1ab. In the vast majority of coronaviruses, this particular site (VKLQ|NNEI in HCoV-229E), differs from all other coronavirus M^pro^ cleavage sites in that Asn (rather than another small residue, such as Ala, Ser, or Gly) occupies the P1’ position (36). Peptide cleavage data obtained in earlier studies suggest that the nsp8|nsp9 site is cleaved less efficiently than other sites, indicating 1) a possible regulatory role of this particular site in the timely coordinated processing of the C-terminal pp1a region or 2) a special role of the conserved nsp9 N terminus in viral replication (37). Our data (Fig. 5*A*) showed that recombinant forms of nsp9 carrying an authentic N-terminal sequence were efficiently NMPylated by nsp12. N-terminal flanking sequences were either removed by cleavage with factor Xa (nsp9-His_6_; *SI Appendix*, Table S1) or by M^pro^-mediated cleavage (nsp7-11-His_6_; Fig. 5*A* and *SI Appendix*, Table S1). Importantly, the uncleaved nsp9-containing precursor nsp7-11-His_6_ was shown to be resistant to NMPylation by nsp12, consistent with our data demonstrating that the nsp9-NMP adduct is formed via the N-terminal primary amine (*SI Appendix*, Fig. S5). To obtain more insight into the NiRAN substrate specificity, we then focused on the adjacent N-terminal residues of nsp9. They are structurally flexible in the absence of other proteins, preventing their detection in structures of nontagged forms of nsp9 (26–28, 38), suggesting that their limited natural variation is due to important sequence-specific (rather than secondary structure-related) functions of the nsp9 N-terminal segment. Ala replacements of conserved residues in this region (Fig. 5 *C* and *D* and *SI Appendix*, Fig. S8) revealed that N3826 is essential for nsp9 NMPylation in vitro, while N3825A and E3827A substitutions resulted in reduced NMPylation, and M3829A and P3830A substitutions did not evidently affect nsp9 NMPylation. Although substitutions of the N-terminal Asn (N3825A, N3825S) had only moderate effects on nsp9 NMPylation and virus replication in cell culture (Fig. 5 *C* and *D*), the deletion of one Asn residue from the N-terminal 3825-NN dipeptide sequence proved to be lethal for the virus, suggesting a requirement for an Asn residue that is preceded by another residue at the very N terminus, preferentially Asn, although replacements with similar residues appear to be partially tolerated (Fig. 5 *B*, *C*, and *D*). We conclude that the 3825-NN dipeptide and, especially, the coronavirus-wide conserved and essential N3826 residue (*SI Appendix*, Fig. S6) ensure the proper binding and orientation of the nsp9 N terminus in the NiRAN active site.

Substitution of the subfamily-wide conserved Glu with Ala (E3827A) retained nsp9 NMPylation in vitro but was lethal for the virus in cell culture (Fig. 5 *C* and *D*), indicating additional functions for this residue, for example, in critical interactions of the (NMPylated or unmodified) nsp9 N terminus with other factors involved in virus replication. None of the nsp9 mutations affected the proteolytic processing of nsp9 or any of the neighboring nsps (39) (*SI Appendix*, Fig. S9), suggesting that the lethal phenotype observed for several nsp9 mutations was not caused by a dysregulated proteolytic processing of the C-terminal pp1a region.

### Possible Functions of NMPylated Forms of nsp9 in Coronavirus Replication.

The above data provide evidence that, following M^pro^-mediated processing of the nsp8|9 cleavage site in pp1a/pp1ab, the N terminus of nsp9 can be UMPylated (or modified with another NMP moiety). Also, the outstanding conservation of the nsp9 N terminus (including a singularly invariant Asn residue in the family *Coronaviridae*) and the reverse-genetics data obtained in this study (Figs. 3*E* and 5*D*) lead us to conclude that the described nsp9 NMPylation is biologically relevant and essential for coronavirus replication. Functional consequences resulting from this modification remain to be studied, for example, with respect to the previously described (nonspecific) RNA-binding activity of (an unmodified form of) nsp9 (26–28). N-terminal NMPylation may also affect interactions of nsp9 with protein or RNA substrates or the formation of different quaternary assemblies that have been observed in structural studies and confirmed to be functionally relevant for coronavirus replication, albeit and notably in the absence of this modification (26–29, 40).

Although the target specificities of coronavirus NiRAN domains remain to be characterized in more detail, our data indicate a narrow protein target specificity for coronavirus NiRAN domains. While the conservation of key active-site residues (8, 16) in NiRAN domains of all nidovirus families strongly support conserved “NMPylator” activities for these proteins, the identity and conservation of substrate-binding pocket residues of this domain remain to be characterized and may vary between different families of the order *Nidovirales*. Likewise, relevant targets remain to be identified for other nidoviruses. They may be remote orthologs of nsp9 or other proteins, given the poor sequence conservation outside the five replicase domains that are universally conserved among nidoviruses (8), including the genomic array between M^pro^ and NiRAN where nsp9 is located in coronaviruses.

Also, we cannot presently exclude the possibility that NiRAN domains have additional (including cellular) targets. In this context, it is worth mentioning that bacterial homologs in this emerging family of protein-NMPylating enzymes (NMPylators) (30, 31) appear to have “master regulator” functions in diverse biological processes, such as cellular stress response and redox homeostasis, by NMPylating multiple cellular proteins to modulate or ablate their downstream activities (22, 33).

In this study (Figs. 2 and 4 and *SI Appendix*, Figs. S3 and S5), we were able to show that nsp12 transfers a UMP (NMP) moiety to a single (conserved) position in nsp9, while other proteins were not modified under the conditions used, supporting a well-defined (rather than relaxed) substrate specificity. In line with this, self-NMPylation activity of nsp12 was very low when compared to N-terminal nsp9 NMPylation, and its detection required much longer autoradiography exposure times, and the use of 10-fold increased nsp12 concentrations. Also, our MS analyses failed to provide evidence for NMPylation of nsp12, suggesting that NiRAN domain self-NMPylation is (at best) a minor activity. It should be noted, however, that other studies provided preliminary evidence that the self-AMPylation status of bacterial NMPylators may control their NMPylation activities toward other protein substrates (22, 33). Thus, additional studies would be desirable to investigate possible functional implications of the self-NMPylation activities reported for EAV nsp9 (16) and coronavirus nsp12 (this study), including the proposed chaperone-like role for the folding of the C-terminal RdRp domain (16).

Previously, several hypotheses on possible downstream functions of nidoviral NiRAN domains were considered, including RNA ligase, RNA 5′-cap guanylyltransferase, and protein-priming activities (16), but none of them could be fully reconciled with the information available at the time without making additional assumptions. The data obtained in this study are most compatible with (but do not prove) an involvement of NiRAN domains in protein-primed initiation of RNA synthesis, while previously considered functions of NiRAN domains in 5′-RNA capping or RNA ligation reactions are not supported by these and other data. Thus, for example, the NiRAN active site is suggested to involve a conserved Asp acting as general base (D252 in *P. syringae* SelO; D4271 in HCoV-229E pp1ab; D208 in SARS-CoV-2 nsp12) (*SI Appendix*, Fig. S2) (17, 22, 33), while catalysis in ATP-dependent RNA ligases and RNA capping enzymes proceeds through a covalent enzyme-(lysyl-N)-NMP intermediate involving an invariant Lys residue (41). Also, the pronounced sequence-based specificity of the coronavirus NiRAN for a conserved protein target and the relaxed specificity for the NTP cosubstrate (with a preference for UTP) argue against NiRAN-mediated capping enzyme or RNA ligase-like functions.

Clearly, extensive additional work is required to verify and, if proved, detail a possible role of nsp9-UMP (nsp9-NMP) in protein-primed RNA synthesis, which would connect several interesting but (so far) isolated observations reported previously. For example, it has been established that the 5′ end of coronavirus minus-strand RNAs starts with an oligo(U) stretch (42, 43). This observation is compatible with the idea that minus-strand RNA synthesis is initiated (“primed”) by binding of a UMPylated form of nsp9 to the poly(A) tail, possibly facilitated by its RNA-binding activity and/or interaction with another RTC protein. The UMP moiety provided by nsp9 could subsequently serve as “primer” for nsp7/8/nsp12-mediated oligouridylylation templated by the 3′-poly(A) tail or another oligo(A)-containing sequence in the genome RNA, similar to mechanisms established for the picornavirus VPg protein (44). It also remains to be studied if the proposed “noncanonical” (protein-primed) initiation of negative-strand RNA synthesis provides a link to observations suggesting that coronavirus negative-strand RNAs have a UMP (rather than UTP) at their 5′ terminus (42), which was suggested to indicate endonucleolytic cleavage by an unknown uridylate-specific endonuclease producing phosphorylated 5′ ends. If confirmed, this nucleolytic activity could help release oligo-UMPylated forms of nsp9 from the 5′ end of the nascent minus strand. A possible role of nsp9 in protein priming would also be compatible with previous reverse-genetics studies that implicated nsp9 (and nsp8) in critical and specific interactions with a conserved *cis*-acting RNA element near the 3′ end of the coronavirus genome (45). In the light of this report, these previous observations could now be revisited and extended by further studies.

In conclusion, our data identify a specific activity for an exclusive nidovirus enzymatic marker that is N-terminally linked to the RdRp. In coronaviruses, this newly identified NiRAN-mediated UMPylator/NMPylator activity is used to transfer a UMP (or, slightly less efficiently, another NMP) moiety to the strongly conserved N terminus of nsp9 in a multicycle reaction that is dependent on Mn^2+^ and the adjacent Asn residue and results in the formation of a (low-energy) phosphoramidate bond with the N-terminal primary amine. The nsp9 target becomes available to NMPylation through M^pro^-mediated cleavage of the nsp8|9 cleavage site, indicating functional coupling between the protease and NiRAN domains and, by extension, the RdRp. The conservation of key residues in both the nsp12 NiRAN active site and the nsp9 target, combined with data obtained for two coronavirus species, including that of SARS-CoV-2, provide strong evidence that nsp9 NMPylation is a conserved feature in coronaviruses and part of a critical step in viral replication. The available data lead us to conclude that a specific role of NMPylated forms of nsp9 in protein-primed RNA synthesis is a plausible scenario for corona- and other nidoviruses, while NiRAN may also target other yet-to-be-identified proteins to regulate virus–host interactions. If confirmed, the involvement of a protein primer in viral RNA synthesis would add to the previously detected sequence affinity in the M^pro^/3CL^pro^ and RdRp domains between coronaviruses and the picornavirus-like supergroup (9), which are now united in the recently established class *Pisoniviricites* (46).

Our data also suggest that the essential, selective, and conserved enzymatic activity identified in this study may be used as a target for antiviral drugs. Compounds interfering with the binding (and subsequent modification) of the conserved nsp9 N terminus in the NiRAN active site may be developed into effective and broadly acting antiviral drugs suitable to treat infections caused by animal and human coronaviruses from different (sub)genera, including SARS-CoV-2 and Middle East respiratory syndrome coronavirus.

## Materials and Methods

### Plasmids, Mutagenesis, and Protein Production in *E. coli*.

The coding sequences of the coronavirus proteins produced in this study were amplified by RT-PCR using RNA isolated from HCoV-229E–infected Huh-7 or SARS-CoV-2–infected Vero E6 cells, respectively, and inserted into pMAL-c2 (New England Biolabs) or pASK3-Ub-CHis_6_ (47) expression vectors (*SI Appendix*, Tables S1 and S2) using standard cloning procedures. Single-codon substitutions were introduced by PCR-based site-directed mutagenesis (48). To produce MBP fusion proteins, *E. coli* TB1 cells were transformed with the appropriate pMAL-c2 plasmid construct (*SI Appendix*, Table S1). Fusion proteins were purified by amylose-affinity chromatography and cleaved with factor Xa. C-terminally His_6_-tagged proteins were subsequently purified by Ni-immobilized metal affinity chromatography (Ni-IMAC) as described previously (49). To produce ubiquitin fusion proteins, *E. coli* TB1 cells were cotransformed with the appropriate pASK3-Ub-CHis_6_ plasmid construct (*SI Appendix*, Tables S1 and S2) and pCGI plasmid DNA encoding the ubiquitin-specific C-terminal hydrolase 1 (Ubp1) (47). C-terminally His_6_-tagged coronavirus proteins were purified as described previously (50).

### NMPylation Assay.

The self-NMPylation assay for HCoV-229E nsp12-His_6_ was performed as described for EAV nsp9 (16). Briefly, nsp12-His_6_ (0.5 µM) was incubated in buffer containing 50 mM 4-(2-hydroxyethyl)-1-piperazineethanesulfonic acid (HEPES)-KOH, pH 8.0, 5 mM dithiothreitol (DTT), 6 mM MnCl_2_, 25 µM the indicated NTP, and 0.17 µM the matching [α^32^-P]NTP (3,000 Ci/mmol; Hartmann Analytic) for 30 min at 30 °C. In all other (standard) NMPylation assays of nsp12-mediated NMPylation of nsp9, the reaction conditions were adjusted as follows: nsp12-His_6_ (0.05 µM) and nsp9-His_6_ (4 µM) were incubated in buffer containing 50 mM HEPES-KOH (pH 8.0), 5 mM DTT, 1 mM MnCl_2_, 25 µM the indicated NTP, and 0.17 µM the matching [α^32^-P]NTP. Following incubation for 10 min at 30 °C, reaction samples were mixed with SDS-PAGE sample buffer: 62.5 mM tris(hydroxymethyl)aminomethane⋅HCl (pH 6.8), 100 mM DTT, 2.5% SDS, 10% glycerol, and 0.005% bromophenol blue. The proteins were denatured by heating for 5 min at 90 °C and separated by 12% SDS-PAGE. Gels were fixed and stained with Coomassie brilliant blue solution (40% methanol, 10% acetic acid, 0.05% Coomassie brilliant blue R-250), destained, and exposed to phosphorimager screens for 20 h (to detect nsp12 self-NMPylation) or (up to) 2 h (to assess nsp9 NMPylation). The screens were scanned using a Typhoon 9200 imager (GE Healthcare), and signal intensities were analyzed with ImageJ.

### Measurement of Intact Protein Masses.

For MS analyses, 1 µM nsp12-His_6_ and 10 µM nsp9 (without hexahistidine tag) (*SI Appendix*, Table S1) and increased concentrations of 500 µM UTP and GTP, respectively, were used in the NMPylation assay. Depending on their concentrations and expected protein masses, 1 to 10 µL of the buffered protein solutions were desalted online using a Waters ACQUITY H-Class HPLC system equipped with a MassPrep column (Waters). Desalted proteins were eluted into the electrospray ionization source of a Synapt G2Si mass spectrometer (Waters) by the following gradient of buffer A (water/0.05% formic acid) and buffer B (acetonitrile/0.045% formic acid) using a column temperature of 60 °C and flow rate of 0.1 mL/min: isocratic elution with 5% A for 2 min, followed by a linear gradient to 95% B within 8 min and holding 95% B for additional 4 min.

Positive ions within the mass range of 500 to 5000 *m*/*z* were detected. Glu-Fibrinopeptide B was measured every 45 s for automatic mass drift correction. Averaged spectra were deconvoluted after baseline subtraction and smoothing using MassLynx instrument software with MaxEnt1 extension.

### Tryptic Digest and Analysis of Resulting Peptides by HPLC MS/MS.

UMPylated HCoV-229E nsp9 was digested by the addition of Sequencing Grade Modified Trypsin (Serva) and incubated at 37 °C overnight. Peptides were desalted and concentrated using Chromabond C18WP spin columns (part no. 730522; Macherey-Nagel). Finally, peptides were dissolved in 25 µL of water with 5% acetonitrile and 0.1% formic acid.

The MS analysis of the samples was performed using an Orbitrap Velos Pro mass spectrometer (Thermo Scientific). An Ultimate nano–rapid separation liquid chromatography–HPLC system (Dionex), equipped with a custom end-fitted 50 cm × 75 µm C18 RP column filled with 2.4-µm beads (Dr. Albin Maisch High Performance LC GmbH) was connected online to the mass spectrometer through a Proxeon nanospray source; 6 µL of the tryptic digest was injected onto a 300 µm internal diameter × 1 cm C18 PepMap preconcentration column (Thermo Scientific). Automated trapping and desalting of the sample were performed at a flow rate of 6 µL/min using water/0.05% formic acid as solvent.

Separation of the tryptic peptides was achieved with the following gradient of water/0.05% formic acid (solvent A) and 80% acetonitrile/0.045% formic acid (solvent B) at a flow rate of 300 nL/min: 4% B for 5 min, followed by a linear gradient to 45% B within 30 min and a linear increase to 95% solvent B within 5 min. The column was connected to a stainless steel nanoemitter (Proxeon), and the eluent was sprayed directly toward the heated capillary of the mass spectrometer using a potential of 2,300 V. A survey scan with a resolution of 60,000 within the Orbitrap mass analyzer was combined with at least three data-dependent MS/MS scans with a dynamic exclusion for 30 s using either collision-induced dissociation with the linear ion-trap or higher-energy collisional dissociation combined with orbitrap detection at a resolution of 7,500.

Data analysis was performed using Proteome Discoverer 2.4 (Thermo Scientific) with the SEQUEST search engine, Byonic (Protein Metrics), or data were evaluated manually using Xcalibur (Thermo Scientific).

### Propylation of Free Amino Groups.

nsp12-His_6_ (1 µM) and nsp9-His_6_ (10 μM) were incubated with 500 µM UTP in the NMPylation assay. Subsequently, nsp9-His_6_ was separated by Ni-IMAC, and imidazole was removed from the eluate using a PD-10 desalting column (GE Healthcare). Desalted and dried tryptic peptides (digestion protocol as described in *Tryptic Digest and Analysis of Resulting Peptides by HPLC MS/MS*) of uridylated nsp9-His_6_ were resuspended using a mixture of 25 µL 200 mM sodium phosphate buffer at pH 6, 12.5 µL of sodium cyanoborohydride (NaCNBH_3_; Sigma-Aldrich), 2.5 µL of acetone, and 10 µL of water. The reaction mixture was incubated for 11 h at room temperature. Subsequently, 20 µL 5% trifluoroacetic acid and 20 µL 5% formic acid were added. Finally, modified peptides were desalted and concentrated using Chromabond C18WP spin columns (part no. 730522; Macherey-Nagel) and dissolved in 25 µL water with 5% acetonitrile and 0.1% formic acid. Measurement of samples was performed as described in *Tryptic* *Digest and* *Analysis of* *Resulting* *Peptides by HPLC MS/MS*.

### M^pro^ Cleavage Assay.

The protease cleavage reaction was performed for 3 h at room temperature in NMPylation buffer (50 mM HEPES-KOH [pH 8.0], 5 mM DTT, 1 mM MnCl_2_, and 25 µM UTP) containing 5 µM nsp7-11 protein construct (or mutant derivatives thereof) and 4.5 µM nsp5 (*SI Appendix*, Table S1). The reaction was stopped by the addition of SDS-PAGE sample buffer. After heating at 90 °C for 5 min, the proteins were separated by 12% SDS-PAGE and stained with Coomassie brilliant blue.

### Cells and Viruses.

HCoV-229E wild type and mutants were propagated and titrated by plaque assay in Huh-7 cells as described previously (51). Recombinant vaccinia viruses derived from vHCoV-inf-1 were propagated in CV-1, BHK-21, and D980R cells as described previously (24, 52).

### Mutagenesis of HCoV-229E Full-Length cDNA Clones and Plaque Assay.

A list of HCoV-229E mutants generated in this study is provided in the *SI Appendix*, Table S3. HCoV-229E mutants were generated using the recombinant vaccinia virus vHCoV-inf-1, which contains a full-length cDNA of the HCoV-229E genome (NC_002645) as described by Thiel et al. (24). Site-directed mutagenesis of the HCoV-229E cDNA insert was done using previously described methods (24, 52). To generate vHCoV-229E nsp9 mutants, a vHCoV-inf-1 derivative was used in which HCoV-229E nucleotides (nts) 11180 to 11761 were replaced with the *gpt* gene (vRec8). To generate vHCoV-229E nsp12 mutants, a vHCoV-inf-1 derivative was used in which HCoV-229E nts 12497 to 15276 were replaced with the *gpt* gene (vnsp12GPT). vRec8 and vnsp12GPT, respectively, were used to produce the desired vHCoV-229E mutants by homologous recombination with appropriate plasmid constructs derived from pJet1.2-nsp7-10 (containing HCoV-229E nts 11151 to 12689) or pJet1.2-nsp9-13 (containing HCoV-229E nts 12010 to 15780) and containing the desired mutations in the nsp9 or nsp12 coding sequence. 5′-Capped HCoV-229E genome-length RNAs transcribed in vitro from purified genomic DNA of the respective recombinant vHCoV-inf-1–derived vaccinia viruses were used to transfect Huh-7 cells using previously described protocols (24, 52). At 72 h posttransfection, cell culture supernatants were collected (passage p0) and 5 × 10^5^ Huh-7 cells were inoculated with 500 µL of p0. At 1 h postinfection (p.i.), the virus inoculum was replaced with 1 mL of fresh medium. Virus titers in cell culture supernatants obtained at 24 h p.i. (p1) were determined by plaque assay. All transfection experiments were done in triplicate using at least two different preparations of in vitro-transcribed RNA.

### MSA.

An in-house MSA of >2,500 full genome sequences of the family *Coronaviridae* was used in the Viralis platform (53) to prepare a protein MSA of 48 viruses, representing 48 species delineated by DEmARC (54), 46 established (7), and 2 pending. The MSA encompassed nsp9 along with the C-terminal residue of nsp8 and the N-terminal residue of nsp10 that are part of the nsp8|nsp9 and nsp9|nsp10 cleavage sites, respectively.

### Note.

While our manuscript was under review, a preprint of a study by Yan et al. was published reporting the cryoelectron microscopic structure of a SARS-CoV-2 nsp7/8/9/12/13 complex (55). In this structure, the SARS-CoV-2 nsp9 N terminus was found to be bound in the NiRAN active site, involving critical interactions with the Asn-2 residue (corresponding to N3826 in HCoV-229E). This is in perfect agreement with conclusions drawn from our biochemical study focusing on HCoV-229E and SARS-CoV-2 nsp12 and nsp9. However, while our data provide evidence for a multiple-turnover NMPylation of the N terminus of nsp9 molecules by the nsp12 NiRAN domain, implying that the nsp9 N terminus is temporarily bound in the NiRAN active site, Yan et al. failed to detect NMPylation of the nsp9 N terminus, even though the protein’s N terminus and the nucleotide cosubstrate were found to be suitably positioned in the NiRAN active site to potentially allow formation of a covalent bond between GMP and the nsp9 N terminus. Because of the observed binding involving multiple conserved interactions and the lack of evidence for NiRAN-mediated GMPylation of the nsp9 N terminus, Yan et al. went on to suggest that the nsp9 N terminus acts as an inhibitor (rather than substrate) of a NiRAN-mediated (RNA-specific) GMPylation activity. Our data do not support this idea but lead us to suggest that failure to detect nsp9 GMPylation in that study likely resulted from the use of an nsp9 construct containing (at least) two additional N-terminal residues (Gly-Ser), which is predicted to prevent N-terminal GMPylation of nsp9. Our data (Fig. 5 and *SI Appendix*, Fig. S7) demonstrate that 1) a free N terminus with Asn at position 2 of the nsp9 target is essential for NiRAN-mediated NMPylation of the nsp9 N terminus and 2) the presence of a single additional residue at the N terminus or removal of a single residue from the authentic N terminus abolishes N-terminal modification by NiRAN.

## Supplementary Material

Supplementary File

## Data Availability

Study data are provided in the text and supplemental information.
