# Supplementary material for: Coronavirus replication–transcription complex: Vital and selective NMPylation of a conserved site in nsp9 by the NiRAN-RdRp subunit
Source: Proc Natl Acad Sci U S A. 2021 Jan 20;118(6):e2022310118. doi: 10.1073/pnas.2022310118 (PMC8017715; doi:10.1073/pnas.2022310118)
Supplement: Supplementary File [file pnas.2022310118.sapp.pdf]

PNAS
www.pnas.org

Supplementary Information for

Coronavirus replication-transcription complex: vital and selective NMPylation of a conserved site in nsp9 by the NiRAN-RdRp subunit

Heiko Slanina, Ramakanth Madhugiri, Ganesh Bylapudi, Karin Schultheiß, Nadja Karl, Anastasia Gulyaeva, Alexander E. Gorbalenya, Uwe Linne, John Ziebuhr

Corresponding author: John Ziebuhr
E-mail: john.ziebuhr@viro.med.uni-giessen.de

This PDF file includes:

Figures S1 to S10
Tables S1 to S4
SI References

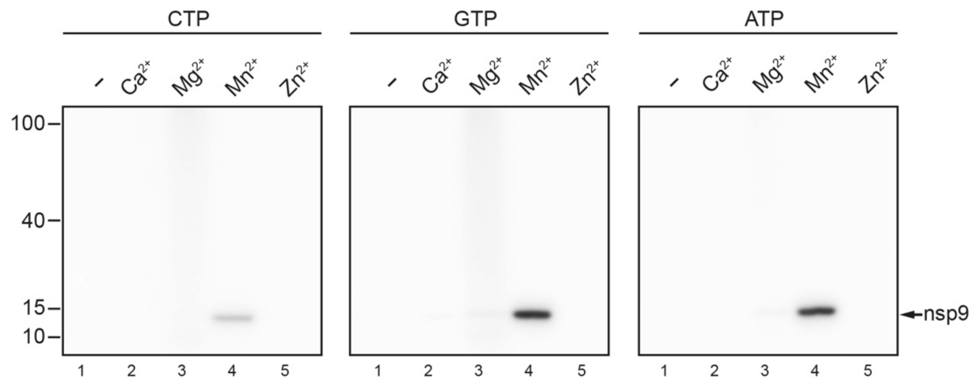

Fig. S1. Metal ion requirements for nsp12-mediated nsp9-His$_6$ NMPylation. NMPylation reactions were supplemented with the indicated [α-^{32}P]-NTP and were performed in the presence or absence of divalent metal ions (at 1 mM) as indicated. Positions of protein markers with molecular masses (in kDa) are indicated to the left.

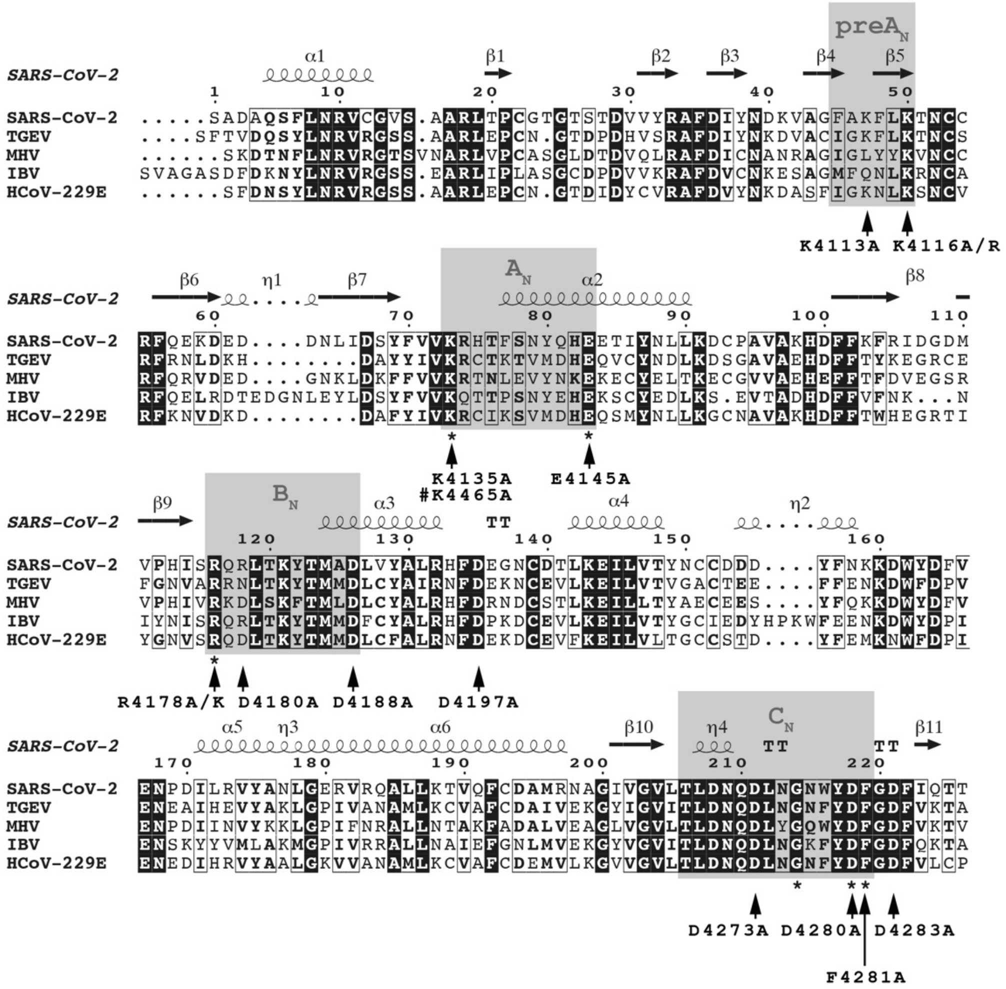

Fig. S2. Sequence alignment of NiRAN domains of five coronaviruses representing the genera *Alpha-*, *Beta-* and *Gammacoronavirus* of the family *Coronaviridae*. The alignment was generated using Clustal Omega (1) and rendered using ESPript version 3.0 (2). Amino acid substitutions in HCoV-229E nsp12 and SARS-CoV-2 nsp12 (#) characterized in this study are indicated by arrowheads. Residue numbering is according to their position in pp1ab. NiRAN sequence motifs (preA$_N$, A$_N$, B$_N$, C$_N$) conserved in members of the order *Nidovirales* (3, 4) are highlighted by gray boxes. Residues conserved in all nidovirus families (4) are indicated by asterisks. Secondary structure elements of SARS-CoV-2 nsp12 (pdb 6XEZ) are shown above the alignment, with residues numbered according to their position in nsp12. Abbreviations used: SARS-CoV-2, severe acute respiratory syndrome coronavirus 2 (NCBI NC_045512.2); TGEV, porcine transmissible gastroenteritis virus, strain Purdue (UniProt P0C6Y5); MHV, murine hepatitis virus, strain A59 (GenBank AGT17724.1), IBV, avian infectious bronchitis virus, strain Beaudette (NCBI NP_00134.1); HCoV-229E, human coronavirus 229E (NC_002645).

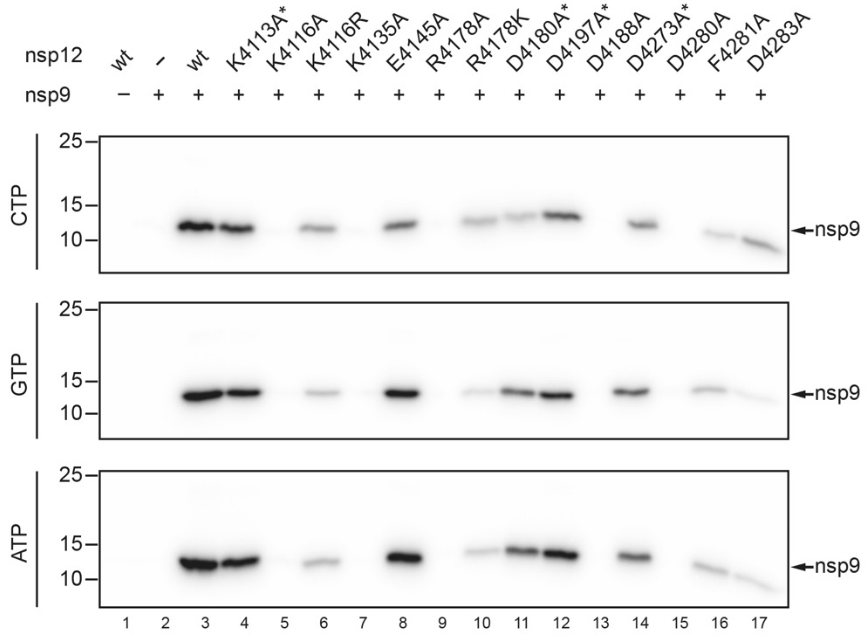

Fig. S3. CMPylation, GMPylation, and AMPylation of nsp9 by recombinant HCoV-229E nsp12 proteins with amino acid replacements in the NiRAN domain. NMPylated nsp9-His$_6$ produced by the different mutant forms of nsp12-His$_6$ in the presence of [α-^{32}P]-CTP, [α-^{32}P]-GTP, or [α-^{32}P]-UTP was analyzed by SDS-PAGE and phosphorimaging (for details, see Materials and Methods and the legend to Fig. 3D).

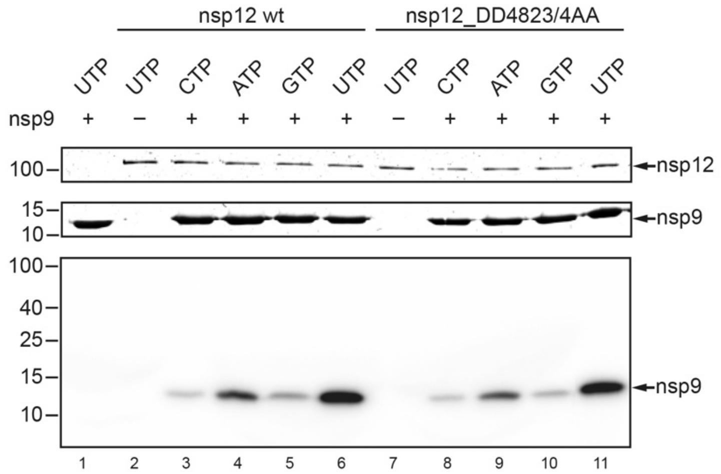

Fig. S4. HCoV-229E nsp12-mediated NMPylation of nsp9 in the presence of different nucleotides and the presence or absence of a catalytically active RdRp domain. Comparison of nsp9-His_6 NMPylation activities of nsp12-His_6 (wt, lanes 1-6) and a mutant form of nsp12-His_6 carrying an Asp-Asp dipeptide replacement (DD4823/4AA) in polymerase motif C (lanes 7-11) in the presence of different [α-^{32}P]-NTPs as indicated. NMPylation reactions were performed as described in Materials and Methods. Reactions mixtures were separated by SDS-PAGE and stained with Coomassie brilliant blue (top panels). Radiolabeled proteins were visualized by phosphorimaging of the same gel (bottom panel). Sizes (in kDa) of molecular mass markers used in this experiment are indicated to the left and Coomassie blue-stained and radiolabeled proteins, respectively, are indicated to the right.

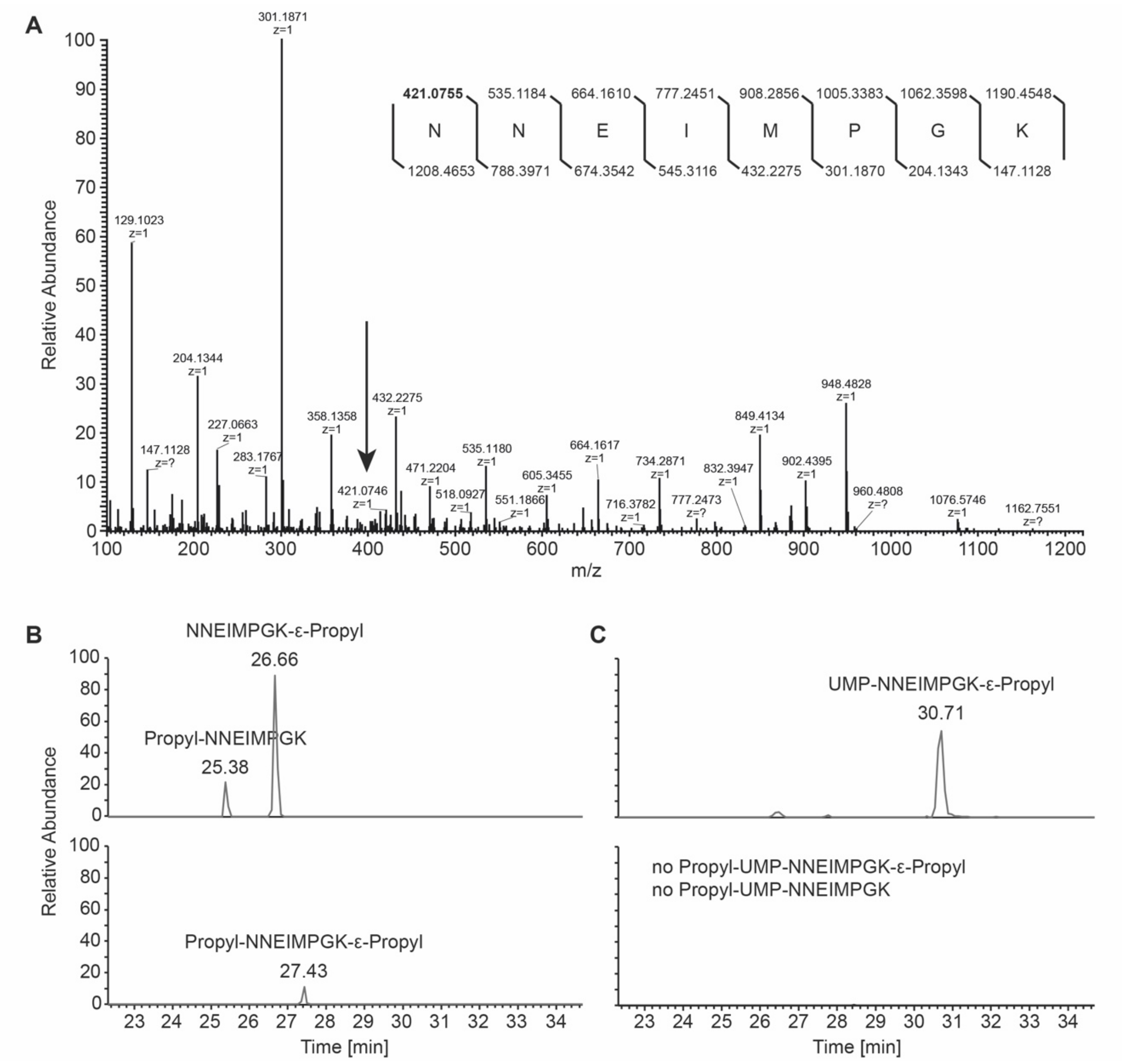

Fig. S5. Covalent NMP-binding at the N-terminal primary amine group of nsp9. (A) Tandem mass spectrum of the tryptic N-terminal nsp9 peptide with a covalently bound UMP moiety. Fragment masses observed indicate that the UMP moiety is bound at position 1 (fragment 421.0746 m/z, calc. 421.0756 m/z, marked in bold). The N-terminal peptide (NNEIMPGK) **(B)** and the UMP-modified peptide (UMP-[NNEIMPGK]) **(C)** were modified by propyl-groups using acetone and $NaCNBH_3$. Under the reaction conditions used, acetone modifies free primary amino-groups. **(B)** Shown are the Extracted Ion Chromatograms (EICs) of the peptide modified by one propyl-group (top lane) and the dipropylated variant (bottom lane). Signal identities were verified by tandem mass spectrometry. Panel **(C)** shows the corresponding EICs for the N-terminal nsp9 peptide with a covalently bound UMP moiety. A signal corresponding to an N-terminally propylated UMP-peptide or dipropylated variant was not observed (bottom lane), suggesting that one of the two primary amino groups in the peptide was blocked by bound UMP.

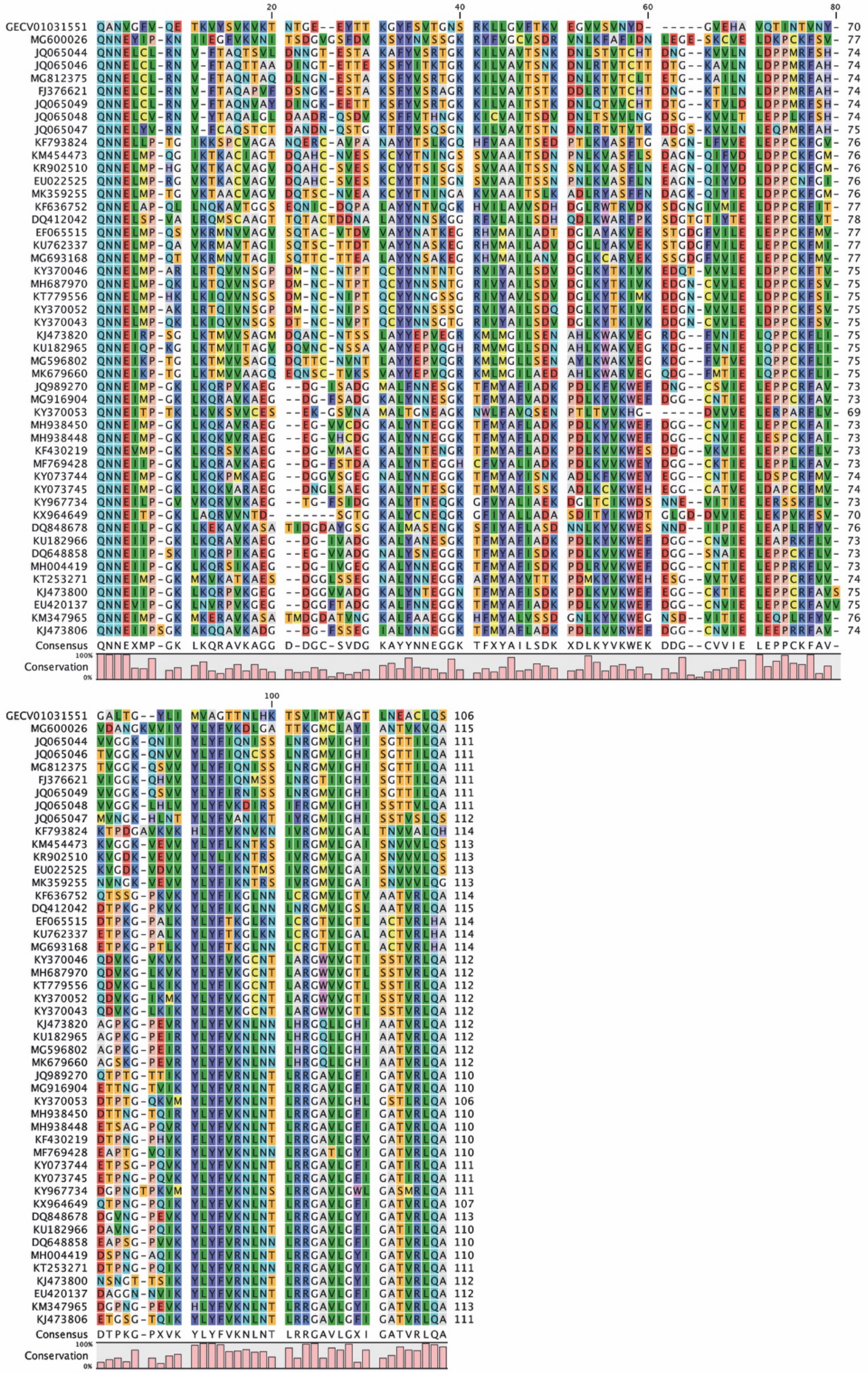

Fig. S6. Alignment of nsp9 sequences representing all virus species in the two subfamilies (*Orthocoronavirinae* and *Letovirinae*) of the family *Coronaviridae*. The MSA was prepared as

described in Materials and Methods and visualized using CLC Genomics Workbench version 8.5.1 (www.qiagenbioinformatics.com/). Viruses are specified using GenBank IDs for the respective genome sequences, one sequence per species. The virus in the top row represents the subfamily *Letovirinae*, all others belong to the subfamily *Orthocoronavirinae*.

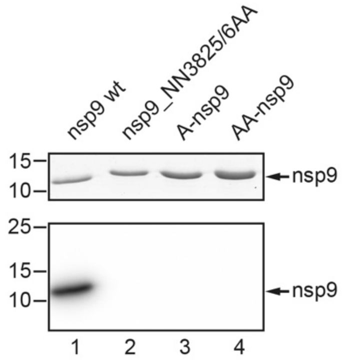

Fig. S7. UMPylation of nsp9 by nsp12 is abolished if the target protein carries one or two additional Ala residue(s) at the N-terminus. Shown are HCoV-229E nsp12-His_6-mediated UMPylation reactions of nsp9-His_6 and three mutant derivatives of nsp9-His_6 (Table S1) carrying replacements of the two N-terminal Asn residues with Ala (nsp9_NN3825/6AA, lane 2), an additional Ala residue at the N-terminus (A-nsp9, lane 3) or two additional Ala residues at the N-terminus (AA-nsp9, lane 4). Reaction products were separated by SDS-PAGE (top panel) and radiolabeled proteins were visualized by phosphorimaging (bottom panel) of the SDS-polyacrylamide gel as described in Materials and Methods. Molecular masses (in kDa) of protein markers are indicated to the left.

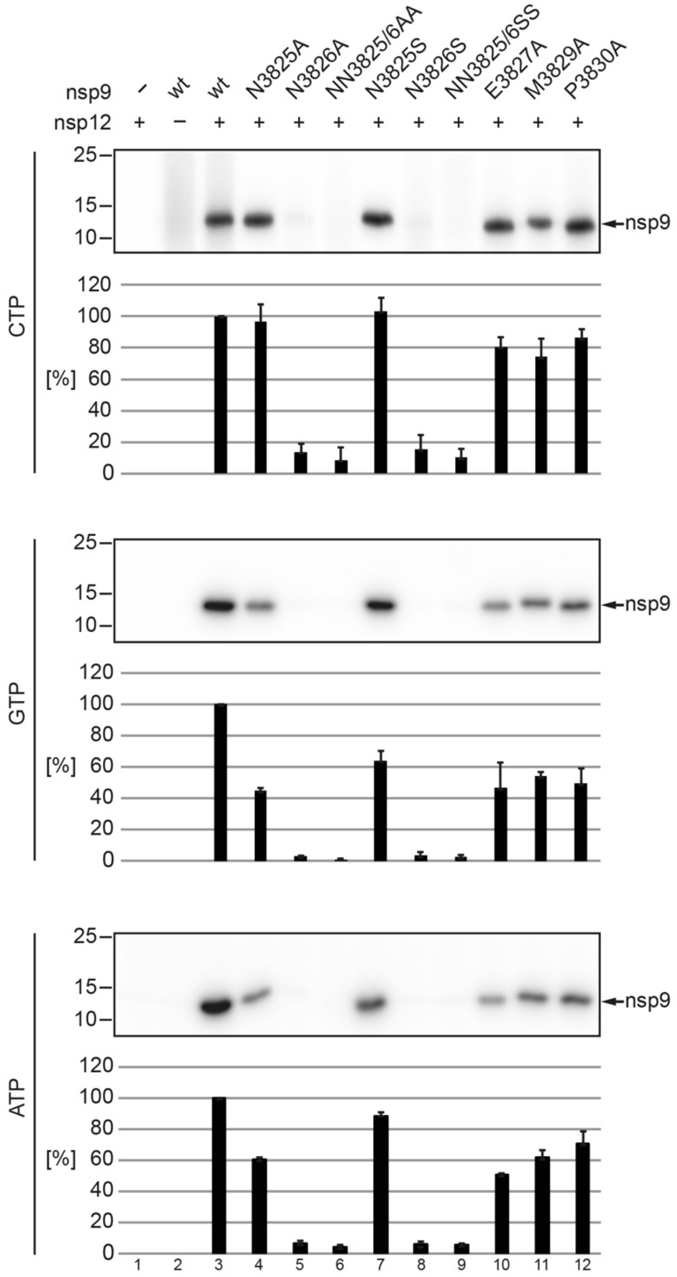

Fig. S8. Mutational analysis of the HCoV-229E nsp9 N-terminus. Nsp12-His_6 mediated NMPylation of wt or mutant forms of nsp9-His_6 carrying amino acid replacements at the protein's N-terminus was determined as described in Materials and Methods and in the presence of different [α-^{32}P]-NTPs as indicated to the left. Reaction mixtures were separated by SDS-PAGE and radiolabeled nsp9-His_6 was detected by phosphorimaging. Molecular masses (in kDa) of protein markers are shown to the left. Below the autoradiograms, relative nsp9 NMPylation activities (means ± SEM) calculated from three independent experiments are given in percent. The signal obtained for nsp9-His_6 wt was used as reference and set to 100%.

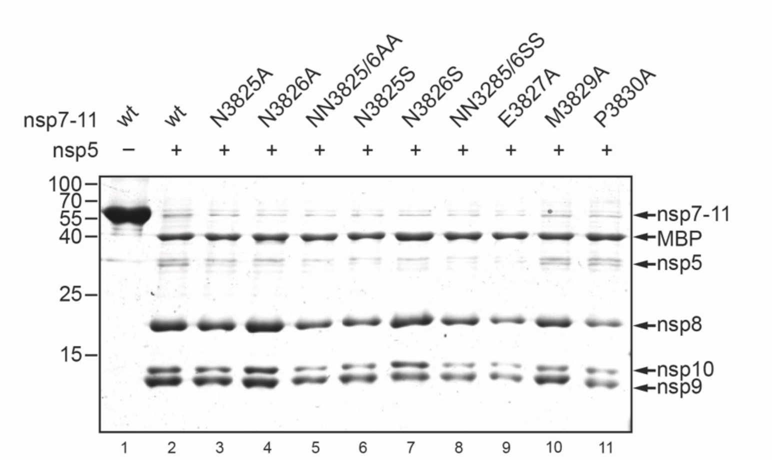

Fig. S9. M^{pro}-mediated proteolytic processing of HCoV-229E nsp7-11 polyprotein constructs containing amino acid replacements at the nsp9 N-terminus. Recombinant wildtype and mutant nsp7-11-His$_6$ proteins produced in *E. coli* were incubated for 60 min at 30°C with recombinant nsp5-His$_6$ (produced from an MBP-nsp5-His$_6$ fusion protein) and reaction products were analyzed in an SDS-polyacrylamide gel stained with Coomassie brilliant blue. Major processing products generated by nsp5-His$_6$-mediated cleavage of the respective nsp7-11-His$_6$ polyprotein constructs are indicated to the right, along with the positions of nsp5-His$_6$ and MBP. Note that nsp7 and nsp11-His$_6$ are not detectable in this gel due to their small sizes (<10 kDa). Molecular masses (in kDa) of protein markers are indicated to the left.

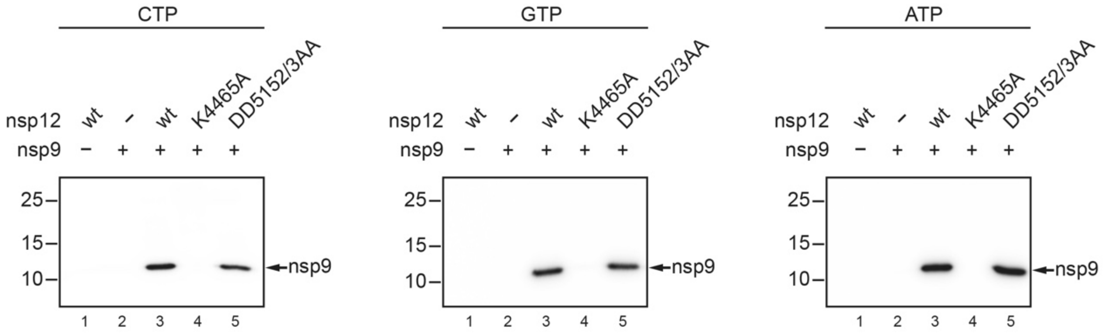

Fig. S10. SARS-CoV-2 nsp12-mediated NMPylation of nsp9. Shown is the SARS-CoV-2 nsp12-His_6 -mediated NMPylation of SARS-CoV-2 nsp9-His_6 using the indicated [α-^{32}P]-NTP. As controls, active-site mutants of the NiRAN (K4465A) and RdRp (DD5152/3AA) domains were used as indicated. Reaction products were separated by SDS-PAGE and radiolabeled proteins were visualized by phosphorimaging as described in Materials and Methods. Shown is an autoradiogram with molecular masses (in kDa) of protein markers indicated to the left.

Table S1. Recombinant HCoV-229E proteins characterized in this study.

HCoV-229E proteins produced in this study	Position in pp1a or pp1ab (NC_002645.1)	Expression plasmid used	Codon change(s)	Corresponding residues in related proteins characterized in this or previous studies (3, 4)
**nsp5-His6**	pp1a 2966-3267	pMAL-c2		
**nsp7-His6**	pp1a 3547-3629	pASK3-Ub-CHis6		
**nsp8-His6**	pp1a 3630-3824	pASK3-Ub-CHis6		
**nsp9**	pp1a 3825-3933	pMAL-c2		
**nsp9-His6**	pp1a 3825-3933	pMAL-c2		
**nsp9-His6_NN3825/6N**	pp1a 3825-3933	pMAL-c2	AAC → ΔAAC	
**nsp9-His6_NN3825/6Δ**	pp1a 3825-3933	pMAL-c2	AACAAT → ΔAACAAT	
**nsp9-His6_N3825A**	pp1a 3825-3933	pMAL-c2	AAC → GCC	
**nsp9-His6_N3826A**	pp1a 3825-3933	pMAL-c2	AAT → GCT	
**nsp9-His6_NN3825/6AA**	pp1a 3825-3933	pMAL-c2	AACAAT → GCCGCT	
**nsp9-His6_N3825S**	pp1a 3825-3933	pMAL-c2	AAC → AGT	
**nsp9-His6_N3826S**	pp1a 3825-3933	pMAL-c2	AAT → AGC	
**nsp9-His6_NN3825/6SS**	pp1a 3825-3933	pMAL-c2	AACAAT → AGTAGC	
**nsp9-His6_E3827A**	pp1a 3825-3933	pMAL-c2	GAA → GCC	
**nsp9-His6_M3829A**	pp1a 3825-3933	pMAL-c2	ATG → GCG	
**nsp9-His6_P3830A**	pp1a 3825-3933	pMAL-c2	CCG → GCG	
**A-nsp9-His6**	A + pp1a 3825-3933	pMAL-c2	+GCG	
**AA-nsp9-His6**	AA + pp1a 3825-3933	pMAL-c2	+GCAGCG	
**nsp10-His6**	pp1a 3934-4068	pASK3-Ub-CHis6		
**nsp7-11-His6**	pp1a 3547-4085	pMAL-c2		
**nsp7-11-His6_N3825A**	pp1a 3547-4085	pMAL-c2	AAC → GCC	
**nsp7-11-His6_N3826A**	pp1a 3547-4085	pMAL-c2	AAT → GCT	
**nsp7-11-His6_NN3825/6AA**	pp1a 3547-4085	pMAL-c2	AACAAT → GCCGCT	
**nsp7-11-His6_N3825S**	pp1a 3547-4085	pMAL-c2	AAC → AGT	
**nsp7-11-His6_N3826S**	pp1a 3547-4085	pMAL-c2	AAT → AGC	
**nsp7-11-His6_NN3825/6SS**	pp1a 3547-4085	pMAL-c2	AACAAT → AGTAGC	
**nsp7-11-His6_E3827A**	pp1a 3547-4085	pMAL-c2	GAA → GCC	
**nsp7-11-His6_M3829A**	pp1a 3547-4085	pMAL-c2	ATG → GCG	
**nsp7-11-His6_P3830A**	pp1a 3547-4085	pMAL-c2	CCG → GCG	
**nsp12-His6**	pp1ab 4069-4995	pASK3-Ub-CHis6		
**nsp12-His6_K4113A**	pp1ab 4069-4995	pASK3-Ub-CHis6	AAA → GCA	
**nsp12-His6_K4116A**	pp1ab 4069-4995	pASK3-Ub-CHis6	AAG → GCG	
**nsp12-His6_K4116R**	pp1ab 4069-4995	pASK3-Ub-CHis6	AAG → AGA	
**nsp12-His6_K4135A**	pp1ab 4069-4995	pASK3-Ub-CHis6	AAA → GCA	**SelO** K113<br>**EAV** nsp9 K94 (pp1a/b K1771)<br>**SARS-CoV** nsp12 K73 (pp1ab K4442)<br>**SARS-CoV-2** nsp12 K4465
**nsp12-His6_E4145A**	pp1ab 4069-4995	pASK3-Ub-CHis6	GAG → GCC	**SelO** E136
**nsp12-His6_R4178A**	pp1ab 4069-4995	pASK3-Ub-CHis6	AGA → GCA	**SelO** R176<br>**EAV** nsp9 R124 (pp1ab R1801)<br>**SARS-CoV** nsp12 R116 (pp1ab R4485)
**nsp12-His6_R4178K**	pp1ab 4069-4995	pASK3-Ub-CHis6	AGA → AAG	**SelO** R176<br>**EAV** nsp9 R124 (pp1ab R1801)<br>**SARS-CoV** nsp12 R116 (pp1ab R4485)
**nsp12-His6_D4180A**	pp1ab 4069-4995	pASK3-Ub-CHis6	GAT → GCA	
**nsp12-His6_D4188A**	pp1ab 4069-4995	pASK3-Ub-CHis6	GAT → GCA	**EAV** nsp9 D132 (pp1ab D1809)<br>**SARS-CoV** nsp12 D126 (pp1ab D4495)
**nsp12-His6_D4197A**	pp1ab 4069-4995	pASK3-Ub-CHis6	GAT → GCA	
**nsp12-His6_D4273A**	pp1ab 4069-4995	pASK3-Ub-CHis6	GAT → GCA	
**nsp12-His6_D4280A**	pp1ab 4069-4995	pASK3-Ub-CHis6	GAC → GCA	**SelO** D262<br>**EAV** nsp9 D165 (pp1ab D1842)<br>**SARS-CoV** nsp12 D218 (pp1ab D4587)
**nsp12-His6_F4281A**	pp1ab 4069-4995	pASK3-Ub-CHis6	TTC → GCC	**SelO** F263<br>**EAV** nsp9 F166 (pp1ab F1843)<br>**SARS-CoV** nsp12 F219 (pp1ab F4588)
**nsp12-His6_D4283A**	pp1ab 4069-4995	pASK3-Ub-CHis6	GAC → GCA	
**nsp12-His6_DD4823/4AA**	pp1ab 4069-4995	pASK3-Ub-CHis6	GATGAT → GCAGCA	**SARS-CoV-2** nsp12 DD5152/3

Table S2. Recombinant SARS-CoV-2 proteins characterized in this study.

SARS-CoV-2 proteins produced in this study	Position in pp1a or pp1ab (NC_045512.2)	Expression plasmid used	Codon changes	Corresponding residues in related proteins characterized in this or previous studies (3, 4)
**nsp12-His$_6$**	pp1ab 4393-5324	pASK3-Ub-CHis$_6$		
**nsp12-His$_6$_K4465A**	pp1ab 4393-5324	pASK3-Ub-CHis$_6$	AAG → GCG	**SelO** K113<br>**EAV** nsp9 K94 (pp1ab K1771)<br>**HCoV-229E** nsp12 K4135<br>**SARS-CoV** nsp12 K73 (pp1ab K4442)
**nsp12-His$_6$_DD5152/3AA**	pp1ab 4393-5324	pASK3-Ub-CHis$_6$	GACGAT → GCCGCT	**HCoV-229E** nsp12 DD4823/4
**nsp9-His$_6$**	pp1a 4141-4253	pASK3-Ub-CHis$_6$		

Table S3. Genetically engineered HCoV-229E mutants characterized in this study.

Recombinant viruses produced in this study	Codon substitution(s)	Corresponding residues in related proteins characterized previously (3, 4)
**HCoV-229E wt**		
**HCoV-229E_nsp9_N3825A**	AAC → GCC	
**HCoV-229E_nsp9_N3826A**	AAT → GCT	
**HCoV-229E_nsp9_NN3825/6AA**	AACAAT → GCCGCT	
**HCoV-229E_nsp9_N3825S**	AAC → AGT	
**HCoV-229E_nsp9_N3826S**	AAT → AGC	
**HCoV-229E_nsp9_NN3825/6SS**	AACAAT → AGTAGC	
**HCoV-229E_nsp9_E3827A**	GAA → GCC	
**HCoV-229E_nsp12_K4113A**	AAA → GCA	
**HCoV-229E_nsp12_K4116A**	AAG → GCG	
**HCoV-229E_nsp12_K4116R**	AAG → AGA	
**HCoV-229E_nsp12_K4135A**	AAA → GCA	**SelO** K113<br>**EAV** nsp9 K94 (pp1ab K1771)<br>**SARS-CoV** nsp12 K73 (pp1ab K4442)<br>**SARS-CoV-2** nsp12 K4465
**HCoV-229E_nsp12_E4145A**	GAG → GCC	**SelO** E136
**HCoV-229E_nsp12_R4178A**	AGA → GCA	**SelO** R176<br>**EAV** nsp9 R124 (pp1ab R1801)<br>**SARS-CoV** nsp12 R116 (pp1ab R4485)
**HCoV-229E_nsp12_R4178K**	AGA → AAG	**SelO** R176<br>**EAV** nsp9 R124 (pp1a/b R1801)<br>**SARS-CoV** nsp12 R116 (pp1ab R4485)
**HCoV-229E_nsp12_D4180A**	GAT → GCA	
**HCoV-229E_nsp12_D4188A**	GAT → GCA	**EAV** nsp9 D132 (pp1a/b D1809)<br>**SARS-CoV** nsp12 D126 (pp1ab D4495)
**HCoV-229E_nsp12_D4197A**	GAT → GCA	
**HCoV-229E_nsp12_D4273A**	GAT → GCA	
**HCoV-229E_nsp12_D4280A**	GAC → GCA	**SelO** D262<br>**EAV** nsp9 D165 (pp1a/b D1842)<br>**SARS-CoV** nsp12 D218 (pp1ab D4587)
**HCoV-229E_nsp12_F4281A**	TTC → GCC	**SelO** F263<br>**EAV** nsp9 F166 (pp1ab F1843)<br>**SARS-CoV** nsp12 F219 (pp1ab F4588)
**HCoV-229E_nsp12_D4283A**	GAC → GCA	
**HCoV-229E_nsp12_DD4823/4AA**	GATGAT → GCAGCA	**SARS-CoV-2** nsp12 DD5152/3

Table S4. Effects of substitutions of conserved HCoV-229E and SARS-CoV-2 NiRAN residues characterized in this study and their presumed functional counterparts in related proteins characterized previously.

**Corresponding residues in proteins characterized in this and previous studies**					**NMPylation activity / virus replication**			
**SelO** (4)	**HCoV-229E**	**SARS-CoV** (3)	**SARS-CoV-2**	**EAV** (3)	**HCoV-229E**	**SARS-CoV** (3)	**SARS-CoV-2**	**EAV** (3)
K113	K4135A	K73A (pp1ab K4442A)	K4465A	K94A (pp1ab K1771A)	– / –	n. d. / –	– / n. d.	– / –
R176	R4178A	R116A (pp1ab R4485A)		R124A (pp1ab R1801A)	– / –	n. d. / –	n. d. / n. d.	– / –
	D4188A	D126A (pp1ab D4495A)		D132A (pp1ab D 1809A)	– / –	n. d. / –	n. d. / n. d.	– / + (reversion)
D262	D4280A	D218A (pp1ab D4587A)		D165A (pp1a/b D1842A)	– / –	n. d. / –	n. d. / n. d.	– / + (reversion)
F263	F4281A	F219A (pp1ab F4588A)		F166A (pp1a/b F1843A)	+ / +	n. d. / +	n. d. / n. d.	– / –

SI References

1. F. Sievers, A. Wilm, D. Dineen, T. J. Gibson, K. Karplus, W. Li, R. Lopez, H. McWilliam, M. Remmert, J. Soding, J. D. Thompson, D. G. Higgins, Fast, scalable generation of high-quality protein multiple sequence alignments using Clustal Omega. *Mol Syst Biol* **7**, 539 (2011).
2. X. Robert, P. Gouet, Deciphering key features in protein structures with the new ENDscript server. *Nucleic Acids Res* **42**, W320-324 (2014).
3. K. C. Lehmann, A. Gulyaeva, J. C. Zevenhoven-Dobbe, G. M. Janssen, M. Ruben, H. S. Overkleeft, P. A. van Veelen, D. V. Samborskiy, A. A. Kravchenko, A. M. Leontovich, I. A. Sidorov, E. J. Snijder, C. C. Posthuma, A. E. Gorbalenya, Discovery of an essential nucleotidylating activity associated with a newly delineated conserved domain in the RNA polymerase-containing protein of all nidoviruses. *Nucleic Acids Res* **43**, 8416-8434 (2015).
4. A. Sreelatha, S. S. Yee, V. A. Lopez, B. C. Park, L. N. Kinch, S. Pilch, K. A. Servage, J. Zhang, J. Jiou, M. Karasiewicz-Urbanska, M. Lobocka, N. V. Grishin, K. Orth, R. Kucharczyk, K. Pawlowski, D. R. Tomchick, V. S. Tagliabracci, Protein AMPylation by an evolutionarily conserved pseudokinase. *Cell* **175**, 809-821 e819 (2018).
